# Molecular Characterization of Membrane Gas Separation under Very High Temperatures and Pressure: Single- and Mixed-Gas CO_2_/CH_4_ and CO_2_/N_2_ Permselectivities in Hybrid Networks

**DOI:** 10.3390/membranes12050526

**Published:** 2022-05-17

**Authors:** Sylvie Neyertz, David Brown, Saman Salimi, Farzaneh Radmanesh, Nieck E. Benes

**Affiliations:** 1 University Savoie Mont Blanc, University Grenoble Alpes, CNRS, Grenoble INP, LEPMI, 38000 Grenoble, France; david.brown@univ-smb.fr (D.B.); saman.salimi@univ-smb.fr (S.S.); 2 Films in Fluids, Department of Science and Technology, MESA+ Institute, University of Twente, P.O. Box 217, 7500 AE Enschede, The Netherlands; f.radmanesh@utwente.nl (F.R.); n.e.benes@utwente.nl (N.E.B.)

**Keywords:** hybrid organic–inorganic membranes, polyOAPS/POSS-imides, gas separation, high temperatures and pressures, molecular dynamics (MD) simulations, grand-canonical Monte Carlo (GCMC) sorption, single-gas and mixed-gas feeds, ideal and real permselectivities

## Abstract

This work illustrates the potential of using atomistic molecular dynamics (MD) and grand-canonical Monte Carlo (GCMC) simulations prior to experiments in order to pre-screen candidate membrane structures for gas separation, under harsh conditions of temperature and pressure. It compares at 300 °C and 400 °C the CO_2_/CH_4_ and CO_2_/N_2_ sieving properties of a series of hybrid networks based on inorganic silsesquioxanes hyper-cross-linked with small organic PMDA or 6FDA imides. The inorganic precursors are the octa(aminopropyl)silsesquioxane (POSS), which degrades above 300 °C, and the octa(aminophenyl)silsesquioxane (OAPS), which has three possible *meta*, *para* or *ortho* isomers and is expected to resist well above 400 °C. As such, the polyPOSS-imide networks were tested at 300 °C only, while the polyOAPS-imide networks were tested at both 300 °C and 400 °C. The feed gas pressure was set to 60 bar in all the simulations. The morphologies and densities of the pure model networks at 300 °C and 400 °C are strongly dependent on their precursors, with the amount of significant free volume ranging from ~2% to ~20%. Since measurements at high temperatures and pressures are difficult to carry out in a laboratory, six isomer-specific polyOAPS-imides and two polyPOSS-imides were simulated in order to assess their N_2_, CH_4_ and CO_2_ permselectivities under such harsh conditions. The models were first analyzed under single-gas conditions, but to be closer to the real processes, the networks that maintained CO_2_/CH_4_ and CO_2_/N_2_ ideal permselectivities above 2 were also tested with binary-gas 90%/10% CH_4_/CO_2_ and N_2_/CO_2_ feeds. At very high temperatures, the single-gas solubility coefficients vary in the same order as their critical temperatures, but the differences between the penetrants are attenuated and the plasticizing effect of CO_2_ is strongly reduced. The single-gas diffusion coefficients correlate well with the amount of available free volume in the matrices. Some OAPS-based networks exhibit a nanoporous behavior, while the others are less permeable and show higher ideal permselectivities. Four of the networks were further tested under mixed-gas conditions. The solubility coefficient improved for CO_2_, while the diffusion selectivity remained similar for the CO_2_/CH_4_ pair and disappeared for the CO_2_/N_2_ pair. The real separation factor is, thus, mostly governed by the solubility. Two polyOAPS-imide networks, i.e., the poly*ortho*OAPS-PMDA and the poly*meta*OAPS-6FDA, seem to be able to maintain their CO_2_/CH_4_ and CO_2_/N_2_ sieving abilities above 2 at 400 °C. These are outstanding performances for polymer-based membranes, and consequently, it is important to be able to produce isomer-specific polyOAPS-imides for use as gas separation membranes under harsh conditions.

## 1. Introduction

Separation processes are an essential part of the chemical industry [1]. The energy costs associated with the traditional separation techniques based on phase changes are very high and the use of more efficient methods is strongly advocated for both economical and environmental reasons [1,2]. Non-porous polymer membranes have emerged as one of the possible alternatives, since they are able to separate small gases and vapors of similar sizes based on their differences in permeabilities [3,4]. Gas separation properties have been studied for a large number of polymers [5], and several membranes, including polyimides, polysulfones, polycarbonates, polyphenylene oxide, cellulose acetate and silicone rubbers, have made it to the commercial stage [6,7].

The use of polymer membranes for the separation of small penetrants is generally restricted to fairly moderate temperatures (typically 25–50 °C) and pressures (typically a few bar), since they tend to lose their structural integrities under harsher conditions. On the other hand, the sieving of hot gases and/or high-pressure feeds require macromolecules that are able to exhibit restricted dynamics at high temperatures [8,9] and/or resistance to penetrant-induced dilation [10]. Therefore, the membranes have to be tested under the same harsh conditions as the processes before the production can be scaled up [11,12,13]. While organic polymers can be cross-linked to improve resistance and limit plasticization [14], inorganic materials usually have much better thermomechanical properties. As such, organic–inorganic hybrids have gained attention for potentially combining the efficient gas separation abilities of the organic moieties, along with the resistance and cost-efficiency of the inorganic moieties [15]. These include both the blends, in which the inorganic phases are physically dispersed in the organic phase [16], and the branched or network structures, in which the inorganic and organic parts are linked through covalent bonding [17].

Cubic polyhedral oligomeric silsesquioxanes (POSS) contain rigid inorganic Si_8_O_12_ cages with organic arms *R* attached to the silicon atoms [18,19,20]. Their dendrite-like protuding *R* are modifiable by conventional chemistry and allow for the POSS to be either blended or cross-linked to other structures [21], among which the gas-sieving polyimides [22,23,24,25]. Due to their versatility, silsesquioxanes can be used in the fields of nanocomposites, electrolytes, liquid crystals, functional coatings or membranes [26]. The latter includes the recently-developed polyPOSS-imides based on amino-functionalized POSS hyper-cross-linked with small dianhydride precursors [27,28,29,30]. These ultrathin default-free membranes are prepared by interfacial polycondensation followed by thermal imidization (Figure S1). They exhibit good gas separation properties because of their organic imide moieties being similar to those in polyimides [31,32], and their hybrid nature allows them to perform under tougher operating temperatures (up to 300 °C) than traditional polymers [27,28,29,33]. In addition, their synthesis can be directly carried out on ceramic disks or hollow fibre supports [34], which give them the potential of being scaled up to membrane modules [33].

**Figure 1:**
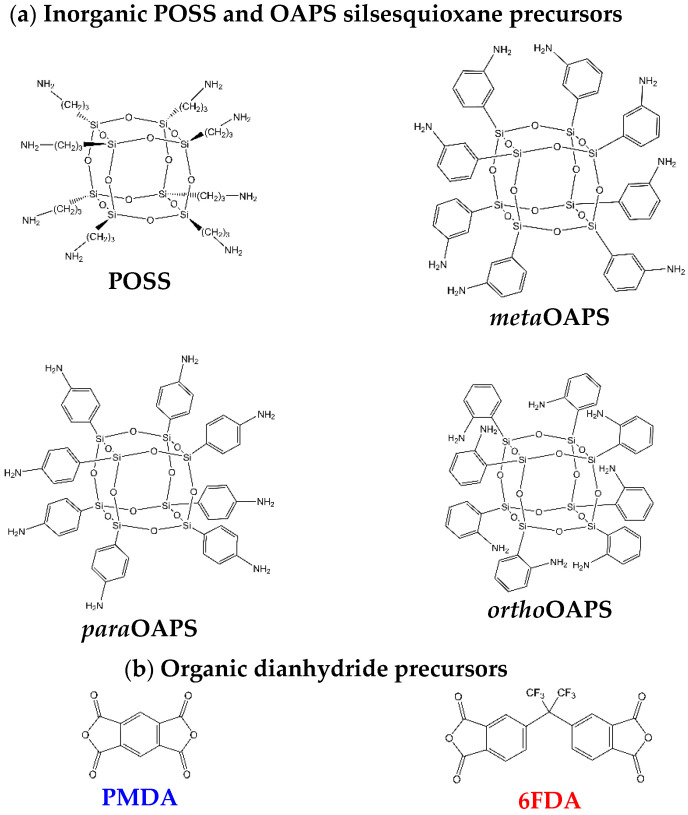
The (**a**) four inorganic and (**b**) two organic precursors used for the polyPOSS-imides and polyOAPS-imides networks under study.

The initial polyPOSS-imide membranes were based on the easily-available octa(aminopropyl)silsesquioxane, i.e., a siloxane cubic cage functionalized with eight -(CH_2_)_3_-NH_2_ arms [27,28,29,30]. It will be referred to hereafter simply as POSS (Figure 1a). The main organic precursors were the pyromellitic dianhydride (PMDA) and the 4,4′-(hexafluoroisopropylidene) diphthalic dianhydride (6FDA) (Figure 1b). However, the flexible aliphatic -(CH_2_)_3_- arms of the POSS were identified as “weak links” in the structures, as they were prone to thermal degradation just above ~300 °C [30,35,36,37].

This work investigates the replacement of POSS with a more thermoresistant precursor, functionalized by amino-substituted phenyl rings, i.e., octa(aminophenyl)silsesquioxane or OAPS (Figure 1a) [19,38]. Indeed, unlike POSS, OAPS-based composites have been shown to resist well above over 400 °C [39,40]. However, this brings out additional difficulties. The first one is that the -NH_2_ group, which is a reactive site for the polycondensation, can be attached in a *meta*, *para* or *ortho* position on the phenyl ring. The second one is that two different routes have been reported for the synthesis of OAPS. The most used scheme involves the nitration of octa(phenyl)silsesquioxane, followed by a reduction and leads to the co-existence of the three isomers. Unfortunately, the respective *meta*:*ortho*:*para* proportions are still ill-defined, since they have been reported by different authors as being 50%:0%:50% [38,41,42,43], 70%:25%:5% [44], 60%:30%:10% [45,46] or 80%:5%:15% [47]. An alternative scheme is the direct synthesis from specific silane precursors [48]. To our knowledge, this route has only been explored once for OAPS, using separately the *meta* and *para* isomers of aminophenyltrialkoxysilanes [49]. It does lead to isomer-specific OAPS, but the cage sizes are not as well controlled as in the nitration/reduction route [38,44,49,50]. The OAPS under study in this paper are based on the silane precursor route, i.e., each of the three isomers is considered separately in its pure form (Figure 1a) in order to clearly assess the effects of the substitution position.

When new materials are being developed, atomistic simulations, such as molecular dynamics (MD) and Monte Carlo (MC) calculations, can be used prior to experiments in order to pre-screen candidate structures under various operating conditions [51,52,53,54]. This is even more critical for high temperatures and pressures, since these are often difficult to implement in a laboratory and potentially hazardous [12,53,55]. In addition, mixed-gas measurements are much more complicated and time-consuming than pure-gas conditions [13,56,57,58]. Within this context, molecular modelling is not aimed at replacing the experiments, but at selecting the most promising structures and avoiding losing time on less interesting ones. It also provides a detailed molecular understanding of the materials and the sorption/diffusion processes, which is difficult to obtain by real experimental analyses. This work illustrates this approach by investigating whether all or some of the polyOAPS-imides membranes are potentially able to maintain their gas separations properties under very harsh conditions and whether more experimental efforts should be devoted to try to better control both aforementioned OAPS synthesis routes.

**Figure 2:**
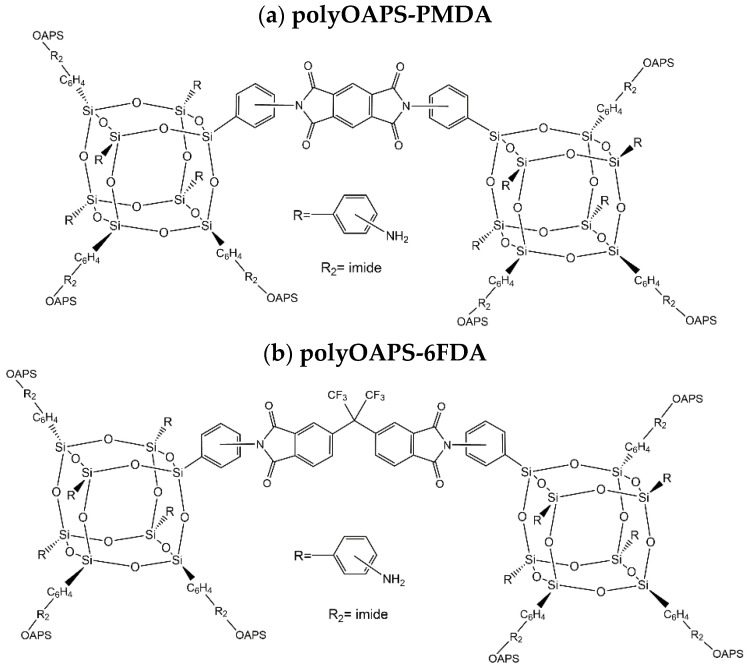
The general chemical formula of polyOAPS-imide networks based on (**a**) the PMDA and (**b**) the 6FDA organic precursors, with an average connectivity of four links *per* OAPS cage.

Figure 2 shows the general chemical formula of polyOAPS-imides based on either PMDA or 6FDA. This is the fourth stage in the MD characterization of gas transport in these networks. The first stage involved modelling polyPOSS-PMDA and polyPOSS-6FDA in the pure state and in the presence of CO_2_ and CH_4_ [35,36,37]. The second stage was a high-temperature screening of twenty-two model polyPOSS-imides and polyOAPS-imides networks [59]. The third stage focused on the CH_4_ and CO_2_ sorption isotherms (0–60 bar) at room temperature for eight of the following systems: a poly*meta*OAPS-PMDA, a poly*para*OAPS-PMDA, a poly*ortho*OAPS-PMDA, a poly*meta*OAPS-6FDA, a poly*para*OAPS-6FDA, a poly*ortho*OAPS-6FDA along with a polyPOSS-PMDA and a polyPOSS-6FDA generated exactly in the same way [60]. Although the optimal precursors in terms of structural and mechanical properties were clearly identified as being *ortho*OAPS and PMDA, respectively, the CO_2_/CH_4_ ideal sorption selectivities at room temperature were found to be quite insensitive to the set of precursors tested [59,60].

In view of their intended applications [11], the same eight systems are here fully tested for their N_2_, CH_4_ and CO_2_ permselectivities under much higher temperatures, i.e., at 300 °C for all of them and at 400 °C for the more thermoresistant polyOAPS-imides. The feed gas pressure is systematically set to 60 bar, both for testing harsh conditions and for statistical reasons, since the number of sorbed gas molecules is expected to decrease significantly at such high temperatures. On the other hand, diffusion will be enhanced, so it is difficult to predict how the gas permeabilities and selectivities will vary. We note that the transport of CH_4_ and CO_2_ at very high pressures and temperatures has already been investigated by MD simulations in amorphous polyethylene [61], but our hybrid networks are expected to behave differently from such a highly-flexible melt-state matrix. All eight models are first studied under single-gas feed conditions. In addition, to be closer to the real operating processes [57,62,63,64,65,66], the networks that maintained a sufficient ideal permselectivity at 300 °C and 400 °C are also tested with feeds corresponding to 90%/10% CH_4_/CO_2_ and N_2_/CO_2_ binary-gas mixtures. This amounted to a total of 54 network+gas simulations, each of which included a sorption phase and a production phase.

The details of the simulations and the methodologies related to the gas solubilities, diffusivities, permeabilities and selectivities are briefly described in Section 2. Section 3 summarizes the structural characteristics of the eight network matrices. Section 4 addresses their N_2_, CH_4_ and CO_2_ transport parameters under single-gas conditions, while those for the binary 90%/10% CH_4_/CO_2_ and N_2_/CO_2_ mixtures are reported in Section 5. All the results presented are at 300 °C and 400 °C and for a pressure of 60 bar. To avoid confusion, please note that when the term “polyOAPS/POSS-imide” is used hereafter, it means both polyOAPS-imides and polyPOSS-imides. Each model contains only one type of inorganic precursor (either one of the three OAPS isomers or POSS) and only one type of organic precursor (either PMDA or 6FDA).

## 2. Models and Methodologies

As mentioned in the Introduction, the molecular models of polyOAPS/POSS-imides have been optimized before, both in the pure state and in the presence of different gases [35,36,37,59,60]. We briefly summarize their main features and outline the specificities associated with the present work.

### 2.1. MD Simulation Parameters

All simulations were carried out using the *gmq* parallel package [67]. The force-field was described by the total potential energy *U_pot_*, which is the sum of three bonded potentials, i.e., the angle-bending, torsional and out-of-plane interactions, along with two non-bonded potentials, i.e., the van der Waals and electrostatic interactions in the following equation (Equation (1)):

(1) $$U_{pot}=\sum_{\theta }U_{bend}\left(\theta \right)+\sum_{\tau }U_{tors}\left(\tau \right)+\sum_{i-planar}U_{oop}\left(i\right)+\sum_{(i,j)nb}U_{vdw}\left(r\right)+\sum_{(i,j)nb}U_{coul}\left(r\right)$$

The atom-types, details of the various terms and force-field parameters to be entered in Equation (1) are provided for the OAPS-based structures in the Supporting Information (Tables S1–S3 and Figure S2) [59], while those for the POSS-based structures have been reported earlier [35,36]. The gas molecules were represented by optimized all-atom models for N_2_ [68], CH_4_ [69] and CO_2_ [70]. The time step was set to Δ*t* = 10^−15^ s in the MD integration algorithm [71], and all the high-frequency modes, i.e., the bond stretches, the hydrogen vibrations and the O=C=O bends for CO_2_ were kept rigid using constraints to ensure equipartition of the kinetic energy [72,73]. For the latter point, we note that there are two major problems for classical simulations of molecules containing high-frequency low-amplitude motions, which are as follows: (a) a much shorter time step would be required to correctly integrate the equations of motion, thus, rendering the calculations considerably more expensive; (b) there is little coupling between such motions and the lower frequency ones, which can lead to severe problems of equipartition of kinetic energy and to cases where the time taken to obtain equipartition is much longer than the duration of the simulations.

The temperature *T* and pressure tensor ***P*** were maintained by loose-coupling close to their required values [74,75]. The MD simulations were run under either constant-volume *NVT* or controlled-pressure-tensor *N**P**T* conditions, which allow for the systems to relax towards their equilibrium sizes and shapes. The average thermodynamic data and configurations were stored every 1 ps and 5 ps, respectively, for post-analyses. The molecular structures were visualized with the VMD software [76].

### 2.2. Generation of the Hyper-Cross-Linked Networks

The networks were built using a fully-atomistic bond-forming/relaxation procedure adapted to cross-linked materials [77,78,79,80,81,82,83,84,85,86,87], in which mixtures of the precursors (Figure 1) are progressively cross-linked using a heuristic distance criterion [86]. The protocol is adapted to be as close as possible to the experimental conditions. In the present case (Figure S1) [27,28,29,30], solutions of both precursors in non-miscible solvents are contacted at room temperature. Polycondensation involving the primary amines on the OAPS (or POSS) arms and the organic anhydrides occurs at the interface between both solvents, and results in a homogeneous polyOAPS(or POSS)-(amic acid) thin film. It is then converted into its polyOAPS(or POSS)-imide final form via thermal imidization at 300 °C, with a loss of one water molecule *per* imidization reaction.

The generation procedure for the polyOAPS/POSS-imide models has been described before [35,36,59]. Initially [35,36], the intermediate polyPOSS-(amic acid) step was explicitly modelled, but it introduced a lot of complexity and it was later shown [59] that the direct transformations of the model mixtures into the final imide forms resulted in similar networks. The optimized procedure is as follows [59]: 3:1 dianhydride:OAPS and dianhydride:POSS mixtures are first prepared at room temperature. The C_ket_…N radial distribution functions between the organic C_ket_ ketone carbons and the inorganic N amine nitrogens are then analyzed. All dianhydrides with the sum of the shortest C_ket_…N distances at either end being less than a *R*_min_ criterion of 7 Å are selected (Figure S3 in the Supporting Information), and their bonds between C_ket_ and their O_anhy_ anhydride oxygen are broken. Two new covalent C_ket_-N bonds are created between each of these dianhydrides and the closest inorganic arms, while both O_anhy_ and the four amine hydrogens are removed. The newly created OAPS(or POSS)-diimide links are energy-minimized towards their equilibrium bond lengths, and the model is thermalized and relaxed up to 10 ps with MD under *NVT* conditions. Following this first cross-linking/relaxation cycle, *R*_min_ is re-calculated for all the unreacted dianhydrides, and those within the *R*_min_ ≤ 7 Å criterion are selected to form more bonds. The process continues in an iterative manner until there are no more possible reactions. At this point, the remaining unreacted dianhydrides are removed from the system. Depending on the precursors, the cross-linking procedures were completed within typical MD simulation times of 1000–5000 ps [59].

The final network sizes were ~30,000–40,000 atoms, i.e., they were large enough to be statistically significant [82,84] and over 99% of their atoms were part of a single continuous network (with the few exceptions being either unreacted inorganic cages or small cage-dianhydride-cage blocks). The siloxane Si-O cages represented ~10% of the total number of atoms. Once cross-linked, the six polyOAPS-imides and the two polyPOSS-imides were further relaxed for 20,000 ps under *N**P**T* conditions (with the on-diagonal components of the required pressure tensor ***P*** being set to 1 bar and its off-diagonal components being set to 0) at the experimental imidization temperature of 300 °C. The six polyOAPS-imides were further heated to 400 °C and relaxed for 5000 ps. These relaxation times allowed for the proper stabilization of the thermodynamic properties [59]. Analyses were carried out over the last 5000 ps at 300 °C and over the last 1000 ps at 400 °C. The polyPOSS-imides were not simulated at 400 °C, as their experimental onset of decomposition is known to be ~350 °C [30].

### 2.3. Modelling Single-Gas and Mixed-Gas Sorption in the Networks

A recent review summarizes the three main molecular modelling approaches for predicting gas sorption in polymer bulk models [88]. All three have also been compared in a large-scale 6FDA-6FpDA polyimide model in contact with either single-gas, binary-gas or ternary-gas reservoirs [52]. The most frequently used is the efficient grand-canonical Monte Carlo (GCMC) method [89], which predicts gas sorption in static pre-prepared configurations. Since it does not take into account the effects of the gas loading on the matrix, it is only applicable to low-plasticizing penetrants or at low uptakes if plasticizing penetrants are being considered [51,88,90,91,92,93,94]. In terms of simulation times, GCMC can be further improved when coupled to the excluded-volume map sampling (EVMS) scheme [95,96,97], which screens out matrix regions of very low insertion probabilities [52]. The second approach is the “iterative pressure TPI-MD” [98,99]. It combines the test particle insertion (TPI) formalism for the insertion of gas molecules [100,101], which can also be associated to EVMS [99,102], with controlled-pressure MD, which naturally allows for the matrix to relax upon penetrant sorption. TPI-MD iteratively estimates the pressure of the external gas reservoir in equilibrium with a fixed number of penetrant gas molecules inserted into the matrix and allows for the prediction of sorption curves over an extended pressure range. However, its main drawback is that it is difficult to apply to mixed-gas cases [52,102]. The third approach combines GCMC for the gas sorption with controlled-pressure MD for the matrix relaxation [51,88,91,93,103,104,105,106,107,108,109]. Several alternating cycles of sorption and relaxation are usually required to adjust the sorbed number of molecules corresponding to the newly relaxed volume; hence, this sorption-relaxation approach can be referred to as the “iterative GCMC-MD” method [52]. As for TPI-MD, it is much more computationally expensive than GCMC on its own. While N_2_ or CH_4_ sorption usually converges within less than five GCMC-MD cycles, the number of iterations at room temperature can increase up to 30–40 for highly-plasticizing gases such as CO_2_. Its advantages over TPI-MD are that the exact feed pressures can be specified and that it is much simpler to extend to mixed-gas feeds [52]. Although TPI-MD and GCMC-MD give identical results [52], the latter will, thus, be used in this work. As pointed out by Anstine et al. [88], the number of studies with such relaxation-allowing techniques is still low when compared with those with fixed frameworks, but they strongly improve the predictive insights in uptake regimes where swelling and plasticization are relevant.

In the present work, iterative GCMC-MD calculations were carried out for the hybrid networks at temperatures *T* of either 300 °C or 400 °C and at a pressure *p* of 60 bar for the following five different feeds: single-gas N_2_, single-gas CH_4_, single-gas CO_2_, binary-gas 90%/10% CH_4_/CO_2_ and binary-gas 90%/10% N_2_/CO_2_. A detailed description of the procedure is provided in Ref. [52]. Briefly, the first step is to obtain for each feed at *T* and *p* under study its gas concentrations *C*_feed_(*p*) and its gas solubilities *S*_feed_(*p*) in the pure gas phase (Supporting Information, Tables S3 and S4). The equilibrium number of sorbed gas molecules in the matrix in contact with a specific feed is then obtained when the chemical potential for the gas in the matrix phase, *μ*_matrix_, is equal to its chemical potential in the feed gas phase, *μ*_feed_ [98,99]. In GCMC [110,111], gas molecules are being exchanged between a virtual gas feed of pressure *p* and a static matrix of fixed volume *V* using Monte Carlo moves to establish this equilibrium between both phases. The probabilities of a gas molecule being moved from one phase to the other are related to the Boltzmann factors for the potential energy changes upon insertion into the target phase [100,101]. We point out that only trial insertion and deletion moves are attempted here. Indeed, rotation and translation moves are equivalent to a deletion and re-insertion and are, thus, redundant. The moves are accepted or refused depending on these probabilities and this process is repeated until detailed balance is obtained, i.e., the flux of gas molecules in both directions is the same. This can be carried out irrespective of the nature of the gas molecules in the feed; hence, it is easily applicable to gas mixtures. For rigid gas molecules, convergence at pressure *p* is obtained when *C*_feed_(*p*) and *S*_feed_(*p*) for each type of penetrant in the feed gas phase are related to its concentration *C*_matrix_(*p*) and its solubility *S*_matrix_(*p*) in the matrix phase through the following equation [37,52,99,102,112]:

(2) $$\frac{C_{\mathrm{matrix}}(p)}{C_{\mathrm{feed}}(p)}=\frac{S_{\mathrm{matrix}}(p)}{S_{\mathrm{feed}}(p)}$$

As noted above, the efficiency of GCMC can be significantly improved by using the EVMS formalism, which avoids trial insertions in unfavorable parts of the matrix [52]. GCMC should also be averaged over several configurations, since the gas solubilities can significantly vary from one configuration to another. An optimal configuration is then chosen at the end of the GCMC cycle and if necessary, its sorbed number of penetrants is adjusted to the average from all the configurations tested. Each GCMC cycle is followed by a controlled-pressure MD phase, which allows for the volume to change following the gas loading predicted by GCMC [91,103,104,105]. Unfortunately, the relaxation of the matrix usually perturbs the equality of the concentration and solubility ratios (Equation (2) and GCMC has to be used again in order to re-adapt the number of sorbed molecules to the newly-relaxed system. A second relaxation run by MD follows and so on. Iterative GCMC-MD cycles have to be repeated until Equation (2) is obeyed both after the GCMC and the MD phases.

In the polyOAPS/POSS-imides under study, the GCMC-MD simulations at 300 °C and 400 °C were initiated from the network configurations already relaxed at both temperatures (Section 2.2). For each network, an initial GCMC phase at 60 bar was carried out over the last 20 configurations. While GCMC trial moves are typically 5–20 million *per* configuration at room temperature (depending on the type of penetrant) [52], the average number of penetrants rises much more rapidly to a plateau value at 300 °C and 400 °C. As such, GCMC simulations of just 2 million trial moves were sufficient to determine the loadings corresponding to 60 bar. This is illustrated for CO_2_ in poly*para*OAPS-PMDA at 300 °C in the Supporting Information (Figure S4). The subsequent MD phase was carried out at the same isotropic pressure of 60 bar. The first MD simulation was run on each network+gas system for 200 ps under *NVT* conditions, followed by 1800 ps under *N**P**T* conditions. Its final 20 configurations were then used for the second GCMC phase. A second MD phase followed, but as the number of penetrants changes much less than at the first iteration, it was shortened to 1000 ps. Since solubility is quite low at 300 °C and 400 °C, convergence was attained in most network + gas models within three to five GCMC-MD iterations, which is considerably less than what is necessary at room temperature [52,60]. Despite the lower solubilities at the higher temperatures, the gas uptakes at 60 bar were still significant enough for the transport parameters to be statistically reliable. The gas solubility coefficients *S*_gas_ were simply obtained from the equilibrium gas concentrations in the matrix at *p* = 60 bar using the following equation:

(3) $$S_{\mathrm{gas}}=\frac{C_{\mathrm{matrix}}(p)}{p}$$

In the case of mixed-gas systems, *p* is replaced in Equation (3) by the partial pressure of the component concerned.

### 2.4. Modelling Gas Diffusion, Permeability and Permselectivity in the Networks

All the MD network simulations that contained each their converged number of penetrants at 60 bar and at either 300 °C or 400 °C were extended to longer times under *N**P**T* conditions. The gas mean square displacements (MSD), averaged over all gas molecules of type *i* and over all possible time origins *t*_0_, were monitored during the runs. The gas diffusion coefficients *D*_gas_ were estimated from the limiting plateau values of the MSD vs 6*t* curves using Einstein’s equation, which is as follows:

(4) $$D_{\mathrm{gas}}=\underset{t\to \infty }{\lim}\;\frac{1}{6t}\;<{\left(\;r_{i}\left(t+t_{0}\right)-r_{i}\left(t_{0}\right)\right)}^{2}>$$

In both single-gas and mixed-gas cases, *D*_gas_ implicitly takes into account the presence of all other gas molecules in the bulk system through the variations in the MSD. However, Equation (4) is only valid within the framework of a long-time Fickian diffusive limit, i.e., when the gas MSD are proportional to *t* [100]. While this is generally difficult to achieve within the MD timescale for glassy matrices at room temperature [113], there are specific techniques, such as the trajectory-extending kinetic Monte Carlo (TEKMC), which can extend the penetrant trajectories based on the actual existing MD runs [114]. This was not necessary here, as diffusion is faster at 300 °C and 400 °C, and indeed, the proportionality between the gas MSD and *t* was obtained for all the cases within MD simulations of 5000–30,000 ps. It should be noted that, in principle, it is possible to apply corrections due to the presence of concentration gradients and net fluxes [115,116,117], but these are small with respect to the errors in establishing the plateau values of MSD/6*t*. Taking this into consideration and the fact that there are no net fluxes nor concentration gradients in these simulations, *D*_gas_ is a reasonable estimation of the intrinsic tendency of the penetrant molecules to diffuse in the dense periodic medium.

The permeability coefficients *P*_gas_ of the penetrants in each network were then evaluated from the product of the diffusion coefficient and the solubility coefficient, using the following equation:

(5) $$P_{\mathrm{gas}}=S_{\mathrm{gas}}\times D_{\mathrm{gas}}$$

The ideal separation factor of gas A over gas B by each network, also called the ideal permselectivity *α*_A/B_, was calculated from the ratio of the permeabilities of both gases under single-gas conditions, using the following equation:

(6) $$\alpha_{A/B}=\frac{P_{\mathrm{gas}-A}}{P_{\mathrm{gas}-B}}=\left(\frac{S_{\mathrm{gas}-A}}{S_{\mathrm{gas}-B}}\right)\times \left(\frac{D_{\mathrm{gas}-A}}{D_{\mathrm{gas}-B}}\right)=\alpha^{S}{}_{A/B}\times \alpha^{D}{}_{A/B}$$

with *α*^S^_A/B_ and *α*^D^_A/B_ being the ideal solubility and the ideal diffusion selectivities, respectively. The ideal permselectivity is reported in most experimental and model studies of gas separation because of the convenience of working with pure gases [52]. However, if actual mixed-gas data are available, Equation (6) can also be used to estimate the real separation factor α^*^_A/B_, the real solubility selectivity *α*^S*^_A/B_ and the real diffusion selectivity *α*^D*^_A/B_. Since the ideal and real transport parameters can differ [13,56,62,63,118,119,120,121], the latter should be closer to the operating process if realistic compositions are being tested. In practice, even if real mixtures are usually very complex [1,52,57], tests on binary mixtures will assess the capacity of the material to separate the key target species. In this work, all the networks were first studied under N_2_, CH_4_ and CO_2_ single-gas conditions, which amounted to a total of 42 simulations. Only the ones that maintained a sufficient ideal permselectivity at 300 °C and 400 °C were tested with the binary mixtures, which amounted to 12 additional simulations.

## 3. The PolyOAPS/POSS-Imide Hyper-Cross-Linked Networks

The structural features of the pure networks have been examined previously [59,60], so we will only outline here their main characteristics. In all the results presented hereafter, the standard errors were calculated using a blocking method from the root mean square (rms) deviations combined with the estimated statistical inefficiency, which stems from the degree of correlation in the MD data [110]. To better distinguish the organic precursors, PMDA-based networks will be indicated in blue and 6FDA-based networks in red in the Figures.

### 3.1. Molecular Connectivities

**Figure 3:**
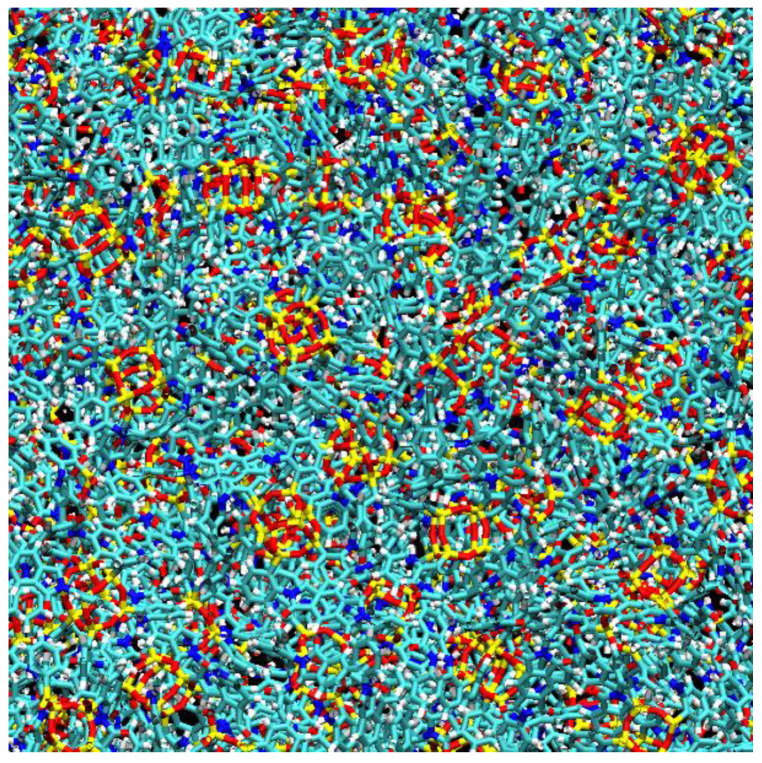
Schematic representation of a (60 Å)^2^ subset of the poly*ortho*OAPS-PMDA network at 400 °C (color code: yellow = Si, red = O, cyan = C, blue = N, green = F; white = H).

The eight-arm nature of the inorganic OAPS and POSS cages gives rise to intricate molecular connectivities when associated to imides (Figure 1 and Figure 2). Three main types of organic–inorganic links have been identified, which are as follows by decreasing order of occurrence [35,36,59,60]: (i) the intercage single-links, in which imides are linked to two different cages, (ii) the intracage links, in which imides are attached to two arms of the same cage and (iii) the intercage double-links, in which two imides are linked to two different arms of the same two cages. Schematic representations of such links are provided in the Supporting Information (Figure S5). The probability density distributions of the number of arms linked *per* cage are Gaussian-like and extend over the full range of possible values (0 to 8 arms linked) (Figure S6), while the corresponding averages vary between 4.5 and 5.4 (Table 1). Experimentally, it has been shown using X-ray photoelectron spectroscopy (XPS) that there can be up to 4.9 links *per* POSS cage for polyPOSS-6FDA upon completion of the polycondensation [29]. This is exactly what we find for the corresponding model network. However, the number of links *per* inorganic cage is not fully representative of the cross-linking densities (i.e., the number of different cages linked to a cage), since some cages have either intracage links, which do not count, or double intercage links, which only count as one for the cross-linking density. As shown by the last line in Table 1, the actual cross-linking densities are ~4.0 for the PMDA and ~3.6 for the 6FDA networks. Energies show no indications of unrelaxed molecular strains [59]. A snapshot of the poly*ortho*OAPS-PMDA system at 400 °C is provided in Figure 3 to better visualize the complexity of such hybrid networks.

A higher cross-linking density is an asset in terms of resistance, and consequently the PMDA networks are expected to have better mechanical properties than the 6FDA networks. This is indeed the case, with the notable exception of the networks based on *ortho*OAPS [59,60]. When associated to the short and rigid PMDA linker, the average intercage Si^…^Si distance is much smaller for the *ortho* (~11.1 Å) than for the *meta* (~15.5 Å) and the *para* (~16.9 Å) isomers. This tends to favor the occurrence of intracage links, and as such, it leads to a low cross-linking density for poly*ortho*OAPS-PMDA. When associated to the more flexible 6FDA linker, the average intercage Si^…^Si distances are closer (~13.7 Å for *ortho*OAPS, ~15.1 Å for *meta*OAPS and ~15.8 Å for *para*OAPS), but there are now significant differences in the angles between the imide N nitrogens and the 6FDA central carbon C. Indeed, the average N^…^C^…^N angle is ~107° in *ortho*OAPS, whereas it is ~98° in *meta*OAPS and ~94° in *para*OAPS. The 6FDA linker adapts to the constrained geometry of the *ortho*OAPS isomer by stretching, which favors intercage links, and in turns, leads to a larger cross-linking density for poly*ortho*OAPS-6FDA than for its PMDA counterpart. This results in the comparative properties of the *ortho*OAPS networks being sometimes in the inverse order than for the other isomers [59,60].

### 3.2. Densities

**Figure 4:**
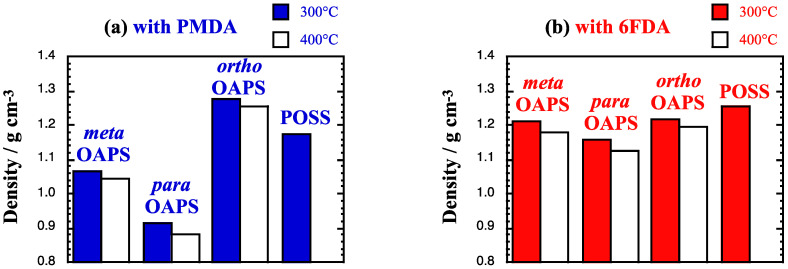
Compared average densities *ρ*_network_ at 300 °C (colored bars) and 400 °C (white bars) for the pure polyOAPS/POSS-imide networks based on the organic (**a**) PMDA and (**b**) 6FDA imides. The pressure is 1 bar and the maximum standard error is 0.002 g cm^−3^.

The relaxed average densities at 300 °C and 400 °C, *ρ*_network_, are displayed in Figure 4.

The density is dependent on both types of precursors with the same trends than at 35 °C [60]. Their cross-linked nature allows for the networks to maintain a rather high cohesion, i.e., *ρ*_network_ of >1 g cm^−3^ (except for poly*para*OAPS-PMDA) at both 300 °C and 400 °C. Indeed, the *ρ*_network_ at 300 °C/400 °C only decrease with respect to 35 °C by ~2.8%/4.5% and ~4.4%/6.2% for the PMDA and 6FDA networks, respectively. This suggests that some of these membranes might be able to conserve gas sieving properties at such high temperatures.

The *ρ*_network_ for the inorganic precursors vary as *para*OAPS < *meta*OAPS < *ortho*OAPS, which is inversely proportional to their molecular dimensions in the pure state [60]. For the organic precursors, they generally vary as PMDA < 6FDA. This is not unexpected, since the flexible 6FDA organic precursor has a good ability to pack and, as such, leads to dense systems. On the other hand, the rigid PMDA precursor cannot adapt as well to the steric constraints introduced by cross-linking and there is more free volume trapped in its networks [30,59,60]. As mentioned above, the exceptions are the *ortho*OAPS systems, with poly*ortho*OAPS-PMDA being denser than poly*ortho*OAPS-6FDA because of the short size and rigidity of both its inorganic and organic precursors.

The model linear thermal expansion coefficients (CTE) [36] lie within the 52–74 10^−6^/°C range, which is in good agreement with the experimental CTE measured in cross-linked polyimide-siloxane films [122,123]. Only the low-density poly*para*OAPS-PMDA has a higher CTE of 90 10^−6^/°C.

### 3.3. Free Volume Available for Gas Insertion

There are several ways to characterize the free volume in molecular simulations of dense matrices [32,93,113,124]. The simplest ones are the geometric approaches, such as the phantom-sphere method, which is based on repeated random trial insertions of a virtual spherical probe of predefined radius [125]. The percentage of probe-accessible volume (%*PAV*) then provides the fraction of the space that can accommodate such a probe. However, purely geometric analyses usually do not take into account the forms of the holes, nor any energetic considerations [60]. The approaches based on geometric and energetic assessments analyze the free volume in a more realistic way [36,37,60,113,126]. This can be achieved by using again the TPI formalism, which randomly inserts specific gas molecules into the matrices and calculates the change in potential energy associated with each insertion, ΔΦ [100,101]. The corresponding probability density distributions *ρ*(ΔΦ), weighted by their Boltzmann factor *ρ_w_*(ΔΦ), are Gaussian [127,128] and provide the range of site energies for penetrant sorption. Examples of *ρ_w_*(ΔΦ) for the polyOAPS/POSS-imides can be found in Ref. [60]. Furthermore, based on the unweighted *ρ*(ΔΦ) and weighted *ρ_w_*(ΔΦ) distributions, the TPI method can estimate the fraction of significant volume (*FSV*), which is associated with the solubility of the target penetrant in the matrix [67]. The integral over *ρ_w_*(ΔΦ) quickly reaches a plateau as the insertion energy increases, and as such, a critical upper limit that accounts for 99.9% of the solubility, ΔΦ_c_, can be defined. The *FSV* is then obtained by the integral of the normalized *ρ*(ΔΦ) up to ΔΦ_c_ as shown by the following equation:

(7) $$FSV=\int_{-\infty }^{\Delta \Phi_{c}}\rho \left(\Delta \Phi \right)d\Delta \Phi$$

In simpler words, the *FSV* is the fraction of insertions associated to energies that contribute to 99.9% of the solubility. When analyzed on the pure matrices, the <*%FSV*> assesses the average percent of the free volume which is available to the specific penetrants under infinite-dilution conditions. They are provided in Table 2 for N_2_, CH_4_ and CO_2_ in the pure polyOAPS/POSS-imides matrices at both 300 °C and 400 °C.

As expected, the various <*%FSV*> at 400 °C are larger than at 300 °C (Table 2), and they correlate negatively with the densities (Figure 4). This confirm that all three OAPS isomers lead to more open networks than the aliphatic-arm POSS, and that there is more free volume available for gas insertion in the PMDA networks. The low-density poly*para*OAPS-PMDA has the maximum <*%FSV*>, due to both its *para*-arms and its PMDA linker being linear. The <*%FSV*> decreases for both other OAPS isomers in agreement with their increasing densities. Similar trends are found in the 6FDA networks with lower <*%FSV*>. Once again, the *ortho*OAPS systems are in reverse order because of their cross-linking specificities (Table 1).

With respect to 35 °C [60], the <%*FSV*> increase by factors of ~1–2 for CH_4_, and ~2–3 for CO_2_, respectively. This leads to the values for different penetrants in the same matrix being fairly similar at high temperatures. At lower temperatures [60], the <%*FSV*> is usually larger for penetrants with low-to-medium solubilities (such as N_2_ and CH_4_), which are mostly governed by the available void-space in the matrix. On the other hand, there is a significant contribution of the interaction energy for more soluble penetrants such as CO_2_. In these cases, the contribution to the <%*FSV*> is mainly restricted to the volume associated to the specific sites providing favorable interactions. When the temperature is higher, the matrices dilate and there are fewer such sites. The <%*FSV*> still varies in the order N_2_ > CH_4_ > CO_2_ for a given matrix, but the actual values are much closer to each other than at lower temperatures.

## 4. Ideal CO_2_/CH_4_ and CO_2_/N_2_ Permselectivities at 300 °C and 400 °C from Single-Gas N_2_, CH_4_ and CO_2_ Uptakes

### 4.1. Single-Gas Solubilities and Diffusivities

Following the iterative GCMC-MD procedure for single-gas sorption, the converged numbers of penetrants at high temperatures and at 60 bar are provided in Table 3 for each network under study. There are clearly enough sorbed penetrants for the results to be statistically significant.

**Figure 5:**
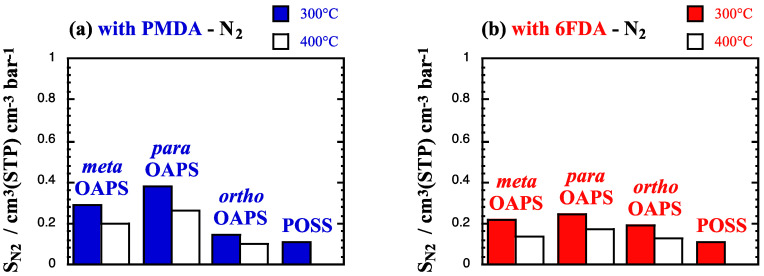

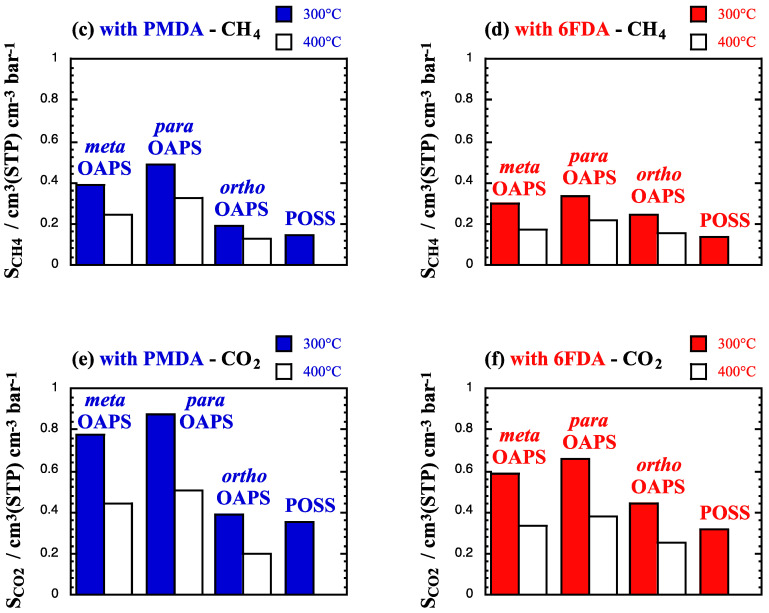
Single-gas solubility coefficients *S*_gas_ at 300 °C and 400 °C in the PMDA and 6FDA polyOAPS/POSS-imides for (**a**,**b**) N_2_, (**c**,**d**) CH_4_ and (**e**,**f**) CO_2_ feeds at 60 bar. The maximum standard error is 0.002 cm^3^(STP) cm^−3^ bar^−1^.

The single-gas solubility coefficients *S*_gas_ (Equation (3) at high temperatures and at 60 bar are provided in cm^3^(STP) cm^−3^ bar^−1^ in Figure 5a,b for N_2_, Figure 5c,d for CH_4_ and Figure 5e,f for CO_2_.

As expected, the *S*_gas_ vary in the same order as the critical temperatures of the penetrants [129], i.e., *S*_N_2__ < *S*_CH_4__ < *S*_CO_2__. Since solubility decreases with temperature, the *S*_gas_ at 400 °C (white bars) are lower than those at 300 °C (colored bars), but the trends as a function of the precursors remain similar to those at 35 °C [60]. In the three polyOAPS-imide networks, the solubilities vary in the order of the available free volume in the pure matrices (Table 2), i.e., *S*_gas_ in *para*OAPS > *meta*OAPS > *ortho*OAPS for the inorganic precursor and *S*_gas_ in PMDA > 6FDA for the organic precursor, except when the latter is associated to the *ortho*OAPS isomer. The initial polyPOSS-imide networks have the lowest available free volumes and as such, the lowest *S*_gas_. Interestingly, they show similar *S*_gas_, regardless of whether POSS is associated with PMDA or to 6FDA and in spite of the latter sorbing more gas molecules (Table 3). This has been shown to be due to a compensation by the difference in volumes [37].

Table 4 reports the percentage of volume swelling upon sorption as a function of the penetrant for each of the model systems.

The changes in volumes are small at both 300 °C and 400 °C and would have been difficult to measure in experimental samples [130,131,132,133]. At low temperatures and high-pressures, non-plasticizing or mildly-plasticizing penetrants, such as N_2_ and CH_4_, usually lead to maximum dilations of 2–3% in glassy matrices [102], while highly-plasticizing penetrants, such as CO_2_, can lead to dilations of up to 10–20% [52,134]. At higher temperatures, the volume swelling remains in the same order, i.e., CO_2_ > CH_4_ > N_2_ [52,60], but the plasticizing effect is clearly strongly reduced because of the decrease in the densities. There are local variations between the various polyOAPS/POSS-imides, which generally correlate with the number of sorbed penetrants (Table 3), e.g., the *para*OAPS and *meta*OAPS isomers tend to swell more than the denser systems. However, these differences remain limited considering the standard errors (maximum 0.3%). In all cases, the reduced amount of swelling is an asset, since it limits the potential decrease in selectivities [119,135].

**Figure 6:**
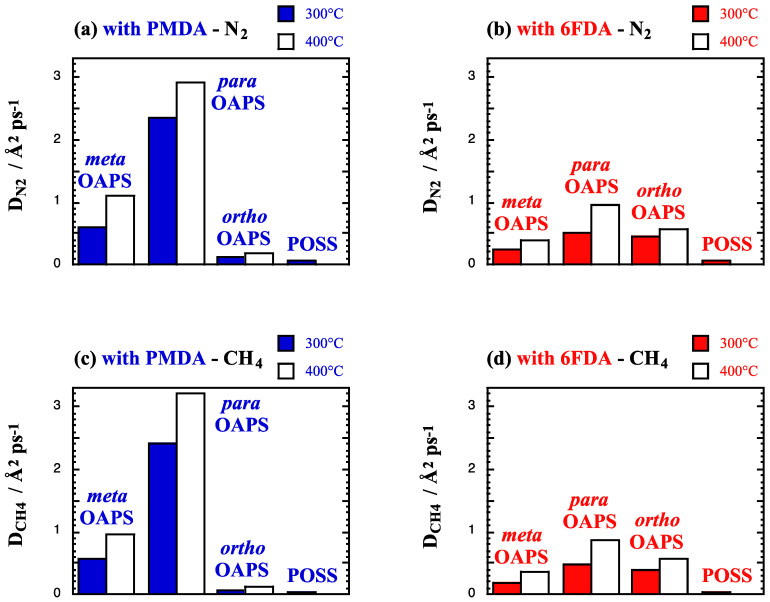

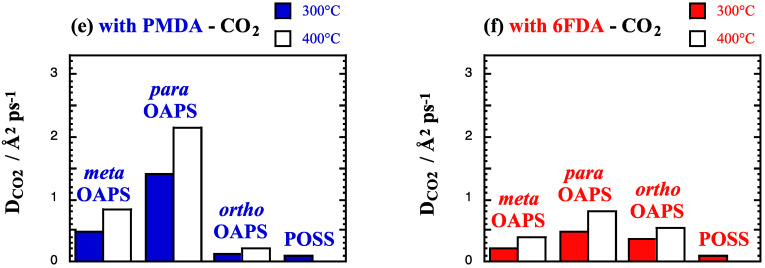
Single-gas diffusion coefficients *D*_gas_ at 300 °C and 400 °C in the PMDA and 6FDA polyOAPS/POSS-imides for (**a**,**b**) N_2_, (**c**,**d**) CH_4_ and (**e**,**f**) CO_2_ feeds at 60 bar. The maximum standard error is 0.1 Å^2^ ps^−1^.

The single-gas diffusion coefficients *D*_gas_ (Equation (4)) at high temperatures and at 60 bar are provided in Å^2^ ps^−1^ in Figure 6a,b for N_2_, Figure 6c,d for CH_4_ and Figure 6e,f for CO_2_.

The differences between the *D*_gas_ at 300 °C (colored bars) and at 400 °C (white bars) confirm that diffusion is significantly enhanced by temperature. The behavior as a function of the gas kinetic diameter [136] observed at lower temperatures in dense glassy polymers [137,138,139,140], i.e., *D*_CO_2__ > *D*_N_2__ > *D*_CH_4__, is only maintained at high temperatures in the systems with the lower available free volumes, i.e., both polyPOSS-imides and the poly*ortho*OAPS-PMDA. The three polyOAPS-6FDA networks have medium amounts of free volume and display similar *D*_gas_ for all three types of penetrants. In the systems with the largest free volumes, i.e., the poly*para*OAPS-PMDA and poly*meta*OAPS-PMDA, the *D*_gas_ follow the order of the Lennard–Jones collision diameters [140] with D_CH_4__ ≈ D_N_2__ > D_CO_2__. The diffusive behavior is, thus, directly linked to the amounts of available free volume in the pure matrices (Table 2). For the same organic precursor, the *D*_gas_ tend to vary in the same direction as *S*_gas_, i.e., a higher solubility leads to a faster diffusion (Figure 5). For the inorganic precursor, diffusion is always faster in the very open *para*OAPS systems and follows the inverse order of the network densities (*D*_PMDA_ > *D*_6FDA_). This is also the case for the *meta*OAPS and *ortho*OAPS systems, although in the latter, poly*ortho*OAPS-PMDA is more dense and consequently *D*_PMDA_ < *D*_6FDA_. Diffusion is always slower in the POSS networks, where there is significantly less free volume available.

### 4.2. Single-Gas Permeabilities and Permselectivities

**Figure 7:**
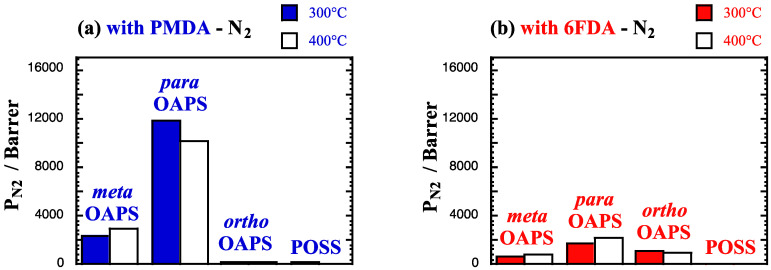

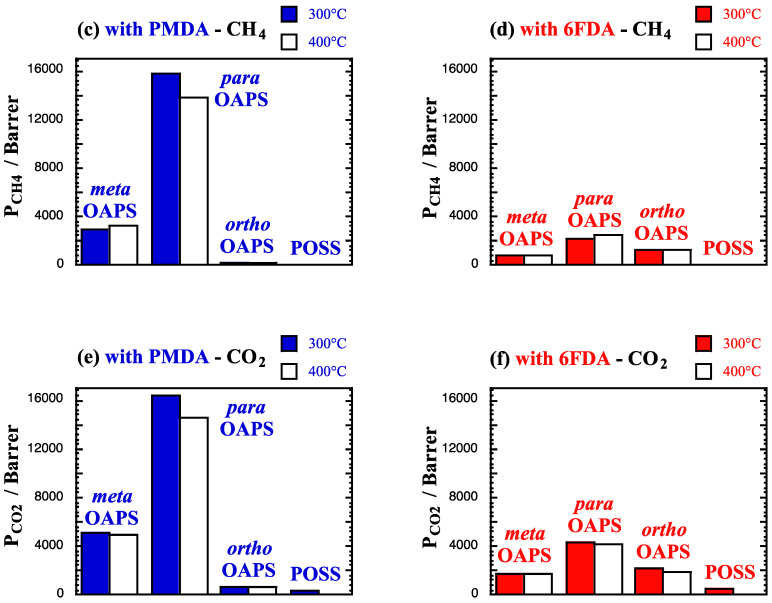
Single-gas permeability coefficients *P*_gas_ at 300 °C and 400 °C in the PMDA and 6FDA polyOAPS/POSS-imides for (**a**,**b**) N_2_, (**c**,**d**) CH_4_ and (**e**,**f**) CO_2_ feeds at 60 bar. The maximum standard error is 470 Barrer for the PMDA networks and 180 Barrer for the 6FDA networks. For POSS, *P*_N_2__ and *P*_CH_4__ are of the order of 80–100 Barrer at 300 °C, and as such, are difficult to observe on the scale of Figure 7.

The single-gas permeability coefficients *P*_gas_ (Equation (5) at high temperatures and at 60 bar are provided in Barrer in Figure 7a,b for N_2_, Figure 7c,d for CH_4_ and Figure 7e,f for CO_2_.

Figure 7 combines for each penetrant under study the thermodynamic effect of its solubility (Figure 5), along with the dynamic effect of its diffusion (Figure 6) in the model polyOAPS/POSS-imides. The higher solubility of CO_2_ seems to offset its slower diffusion and as such, it is more permeable than both other penetrants. The networks can be separated in the following two categories: (1) the high-diffusion and high-solubility poly*para*OAPS-PMDA, poly*meta*OAPS-PMDA and poly*para*OAPS-6FDA, which exhibit a nanoporous behavior and (2) the denser systems, with *P*_gas_ being always higher in the *ortho*OAPS than in the POSS networks and poly*meta*OAPS-6FDA showing an intermediate behavior between both.

**Figure 8:**
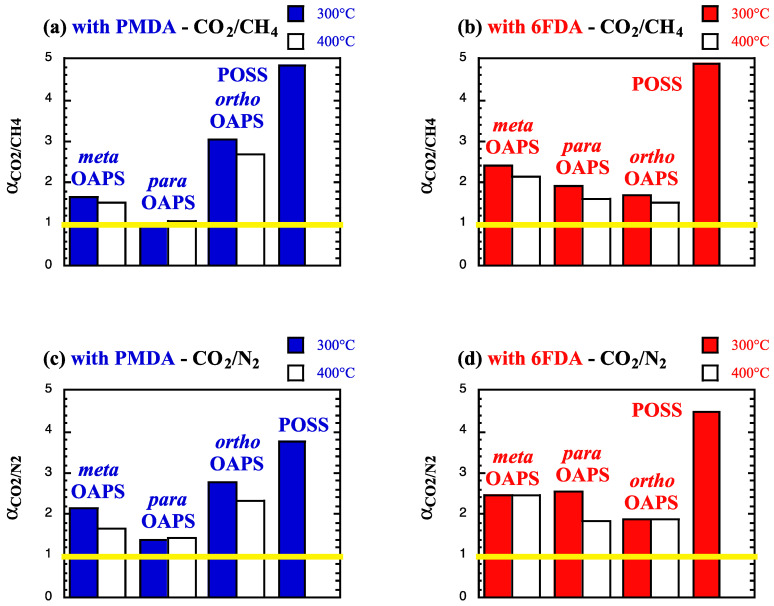
Ideal permselectivities for (**a**,**b**) CO_2_/CH_4_ and (**c**,**d**) CO_2_/N_2_ at 300 °C and 400 °C in the PMDA and 6FDA polyOAPS/POSS-imides calculated from pure feeds at 60 bar. The maximum standard error is 0.2. The yellow lines indicate a *α*_A/B_ of 1.

While it is known that mixed-gas effects vary both with the pressure and the gas composition [63], two classical binary gas mixtures [1,141], i.e., CH_4_/CO_2_ and N_2_/CO_2_ with a ratio of 90%/10%, were examined as test cases. In the selectivities, the more permeable CO_2_ is systematically “gas A”, while CH_4_ or N_2_ are “gas B”. The ideal solubility *α*^S^_A/B_ and diffusion *α*^D^_A/B_ selectivities (Equation (6)) at high temperatures and at 60 bar are provided in Table 5. The ideal permselectivities *α*_A/B_ are displayed in Figure 8. A yellow line corresponding to an ideal permselectivity of 1 is added as a guide to the eye.

As shown by Table 5, the ideal solubility selectivities *α*^S^_A/B_ at 300 °C and 400 °C are always in favor of CO_2_, with the effect being enhanced in the case of N_2_. This is not the case for the ideal diffusion selectivities *α*^D^_A/B_, which reflect the three free-volume-dependent behaviors observed in *D*_gas_ (Figure 6). At such elevated temperatures, the ideal *α*^D^_A/B_ in the networks are lower than the ideal *α*^S^_A/B_, contrary to what happens for most glassy polymers at low temperatures [13]. Indeed, plasticization effects at lower temperature increase the diffusivity of the most soluble penetrants and tend to improve the ideal diffusion selectivity. However, when plasticization is strongly reduced by the temperature, this trend is not followed anymore.

The ideal CO_2_/CH_4_ and CO_2_/N_2_ permselectivities (Figure 8) clearly show that the denser polyPOSS-imides have better sieving properties than the more open polyOAPS-imides [59,60]. However, as explained before, the polyPOSS-imides start degrading above 300 °C [30,35,36,37], while the polyOAPS-imides are expected to resist well over 400 °C [39,40]. Within this context, the most interesting model OAPS-based networks, i.e., those that appear to be able to maintain ideal permselectivities above 2 at 400 °C (white bars in Figure 8), will be further examined for their real separation factor α^*^_A/B_ under mixed-gas conditions. The following four systems were selected from Figure 8: the poly*ortho*OAPS-PMDA and the poly*meta*OAPS-6FDA networks for assessment at both 300 °C and 400 °C. In addition, due to their high ideal permselectivities, the polyPOSS-imides were also tested under mixed-gas conditions, but only at 300 °C.

## 5. Mixed-Gas Separation Factors for Binary 90%/10% CH_4_/CO_2_ and N_2_/CO_2_ Mixtures at 300 °C and 400 °C

### 5.1. Mixed-Gas Solubilities and Diffusivities

Table 6 reports the converged numbers of each penetrant for the four selected networks at 300 °C and 400 °C, following the iterative GCMC-MD procedure for the binary-gas sorption [52] of 90%/10% CH_4_/CO_2_ and N_2_/CO_2_ mixtures at 60 bar. Compared to the pure CH_4_ and N_2_ feeds (Table 3), the uptakes of CH_4_ and N_2_ decrease on average by ~12%, but the total number of penetrants slightly increases because of CO_2_. For the first mixture, the sorbed CH_4_/CO_2_ ratio is ~77%/23% at 300 °C and ~82%/18% at 400 °C. For the second mixture, the sorbed N_2_/CO_2_ ratio is ~70%/30% at 300 °C and ~78%/22% at 400 °C. While CO_2_ does sorb more than its actual percentage in the gas phase, its competitive effect is again very attenuated with respect to lower temperatures. For example, 4% CO_2_ in a ternary 16:8:1 CH_4_/N_2_/CO_2_ gas mixture at 60 bar was found to make up ~40% of the sorbed molecules in a model 6FDA-6FpDA polyimide at 35 °C [52]. Significant sorbed concentrations of CO_2_ have also been reported in other experimental and modelling studies of glassy polymers, even if it was only present at low % in the feed mixtures [62,65,120]. The attenuated effect is confirmed by the differences between 300 °C and 400 °C, with the sorbed ratios at higher temperatures getting closer to the initial 90%/10% composition of the gas mixtures.

**Figure 9:**
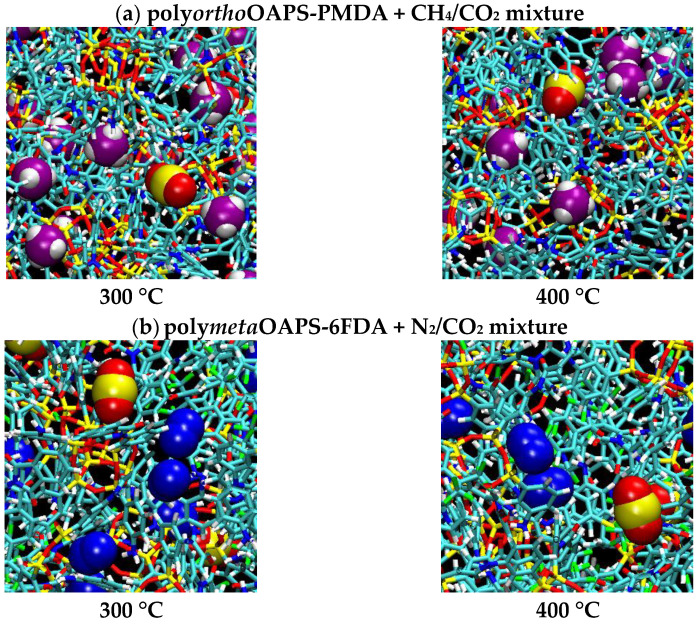
~(20 Å)^2^ close-ups of (**a**) the sorbed CH_4_/CO_2_ mixture in the poly*ortho*OAPS-PMDA matrix and (**b**) the sorbed N_2_/CO_2_ mixture in the poly*meta*OAPS-6FDA matrix at high temperatures and for a mixed-gas pressure of 60 bar. Same color code as Figure 3 for the matrices. For the gases, methane: C = pink, H = white; carbon dioxide: C = yellow, O = red; nitrogen: N = blue.

Figure 9 provides close-ups of the CH_4_/CO_2_ and the N_2_/CO_2_ mixtures sorbed in the poly*ortho*OAPS-PMDA and the poly*meta*OAPS-6FDA matrices.

**Figure 10:**
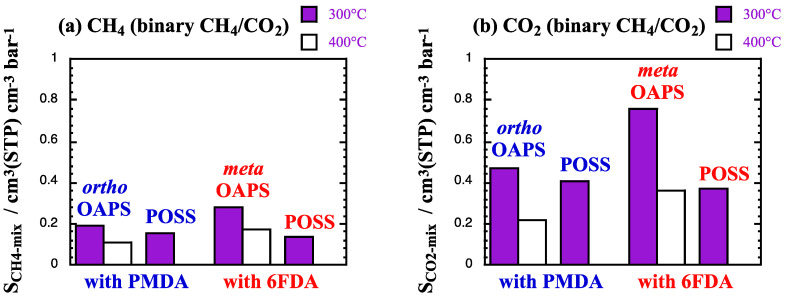

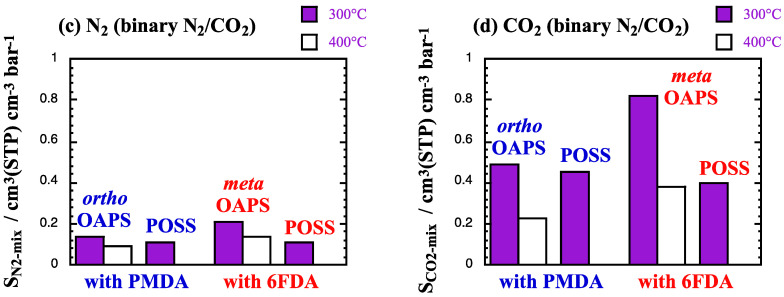
Mixed-gas solubility coefficients *S*_gas-mix_ at 300 °C (purple bars) and 400 °C (white bars) in the selected polyOAPS/POSS-imides for (**a**,**b**) CH_4_ and CO_2_ from the 90%/10% CH_4_/CO_2_ mixture and (**c**,**d**) N_2_ and CO_2_ from the 90%/10% N_2_/CO_2_ mixture at 60 bar. The maximum standard error is 0.001 cm^3^(STP) cm^−3^ bar^−1^.

The mixed-gas solubility coefficients *S*_gas-mix_ are provided in Figure 10 on the same scale as Figure 5. In the following Figures, the results will be presented in the order of both PMDA-based networks, i.e., (1) the *ortho*OAPS at 300 °C and 400 °C and (2) the POSS at 300 °C, followed by both 6FDA-based networks, i.e., (3) the *meta*OAPS at 300 °C and 400 °C and (4) the POSS at 300 °C.

When compared to Figure 5, the *S*_gas-mix_ remain close to their single-gas *S*_gas_ values for the major components, i.e., N_2_ and CH_4_. On the other hand, for the minor component, the *S*_CO_2_-mix_ increase by ~25% at 300 °C and by ~10% at 400 °C compared to the pure CO_2_ feed. This is mostly a consequence of the non-linear behavior of the solubility coefficient with pressure. Due to the concave shape of sorption isotherms, the *S*_CO_2__ of pure CO_2_ at 6 bar is expected to be higher than at 60 bar, which is the limiting lower value over the 0–60 bar range. Under mixed-gas conditions, the uptake of CO_2_ at a partial pressure of 6 bar will be lower than that of pure CO_2_ at 6 bar because of the presence of the less-soluble CH_4_ or N_2_. However, there is less competition compared to pure CO_2_ and consequently, *S*_CO_2_-mix_ in a mixture at 60 bar will remain higher than *S*_CO_2__ at 60 bar. These effects decrease with the solubility as the temperature increases. The percentages of volume swelling (Table 7) are also fairly similar to those found for CH_4_ and N_2_ on their own (Table 4). There is no plasticizing effect with 10% CO_2_ at such high temperatures.

**Figure 11:**
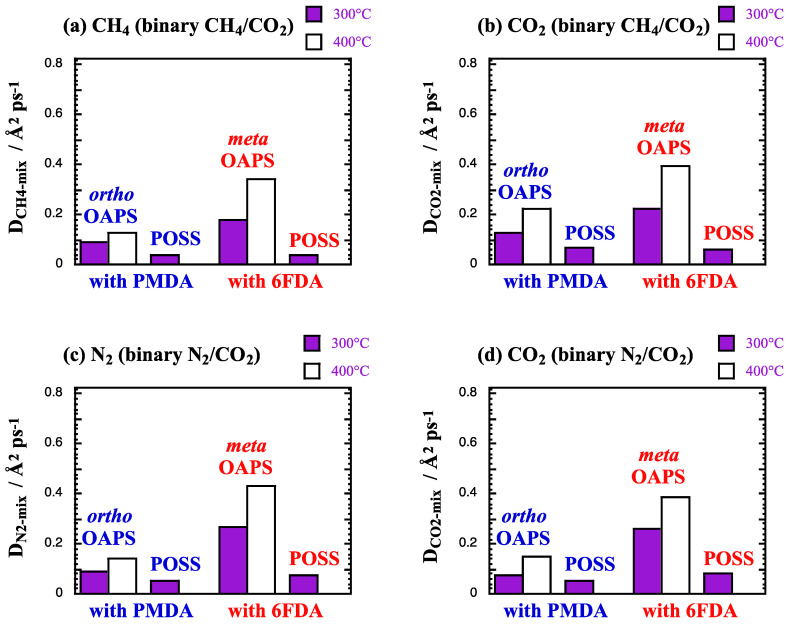
Mixed-gas diffusion coefficients *D*_gas-mix_ at 300 °C and 400 °C in the selected polyOAPS/POSS-imides for (**a**,**b**) CH_4_ and CO_2_ from the 90%/10% CH_4_/CO_2_ mixture and (**c**,**d**) N_2_ and CO_2_ from the 90%/10% N_2_/CO_2_ mixture at 60 bar. The scale is 25% of that in Figure 6. The maximum standard error is 0.03 Å^2^ ps^−1^.

The mixed-gas diffusion coefficients *D*_gas-mix_ are shown in Å^2^ ps^−1^ in Figure 11. The scale is only 25% of that in Figure 6, since the four selected networks had small *D*_gas_ under single-gas conditions.

The *D*_gas-mix_ remain again similar to the single-gas *D*_gas_ (Figure 6). For the CH_4_/CO_2_ mixture, *D*_CH_4_-mix_ ≈ *D*_CH_4__ for all the networks under study. *D*_CO_2_-mix_ ≈ *D*_CO_2__ for the OAPS networks, while it decreases by ~25% for the POSS networks, which swell less upon sorption of the mixture than with the pure CO_2_ feed (Table 4 and Table 7). The kinetic measurements in glassy matrices at lower temperatures show that the presence of CO_2_ enhances CH_4_ diffusion under mixed-gas conditions because of the plasticization effects [13,120,121]. This is not the case at higher temperatures, where the sieving capabilities are barely modified by plasticization. For the N_2_/CO_2_ mixture, *D*_N_2_-mix_ decreases by ~6% and *D*_CO_2_-mix_ by ~18%, and their respective values become very close (Figure 11c,d), i.e., there is no more diffusion selectivity.

### 5.2. Mixed-Gas Permeabilities and Separation Factors

**Figure 12:**
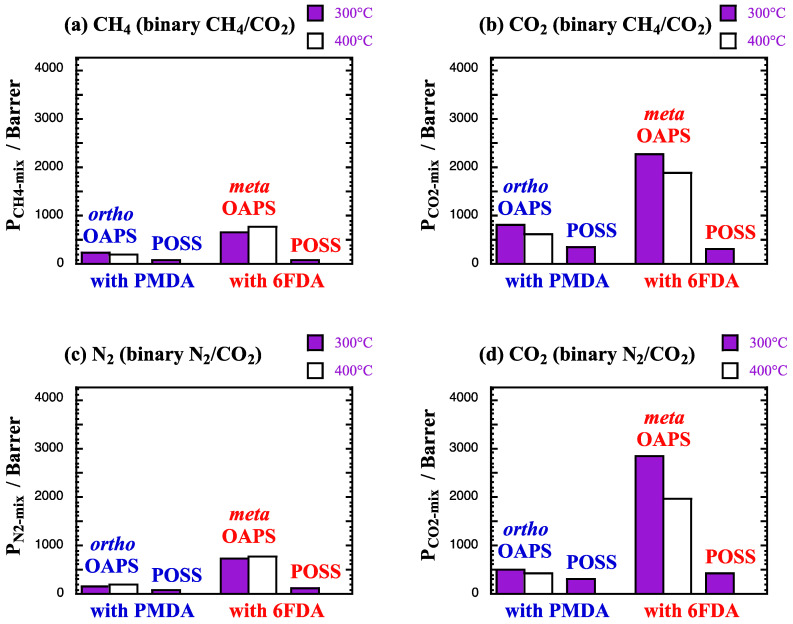
Mixed-gas permeability coefficients *P*_gas-mix_ at 300 °C and 400 °C in the selected polyOAPS/POSS-imides for (**a**,**b**) CH_4_ and CO_2_ from the 90%/10% CH_4_/CO_2_ mixture and (**c**,**d**) N_2_ and CO_2_ from the 90%/10% N_2_/CO_2_ mixture at 60 bar. The scale is 25% of that in Figure 7. The maximum standard error is 55 Barrer for CH_4_ and N_2_ and 125 Barrer for CO_2_.

The mixed-gas permeabilities coefficients *P*_gas-mix_ are provided in Barrer in Figure 12. As for the diffusion, the scale is only 25% of that in Figure 7, since the four selected networks had small *P*_gas_ under single-gas conditions.

The *P*_gas-mix_ reflect the small variations in the solubility and the diffusion coefficients (Figure 10 and Figure 11). In practice, they are slightly lower than the single-gas *P*_gas_ for CH_4_ and N_2_ (Figure 7). On the other hand, the decrease in *D*_CO_2_-mix_ is more than compensated for by the increase in *S*_CO_2_-mix_, and the *P*_CO_2_-mix_ are on average larger than the *P*_CO_2__ by ~10%. Both POSS-based networks at 300 °C have a similar behavior with the lowest permeabilities. The more open OAPS-based structures have larger permeabilities, with the poly*meta*OAPS-6FDA being more permeable than the denser poly*ortho*OAPS-PMDA network, i.e., it varies in the same order than the available free volume (Table 2).

**Figure 13:**
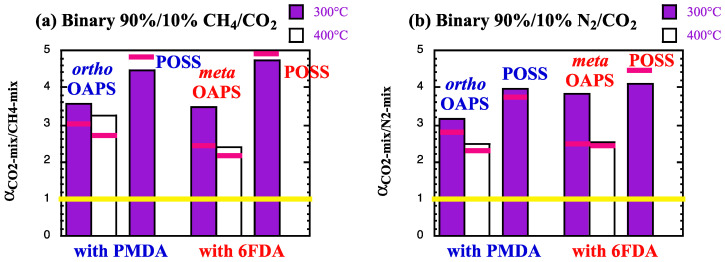
Real (**a**) CO_2_/CH_4_ and (**b**) CO_2_/N_2_ separation factors at 300 °C and 400 °C in the selected polyOAPS/POSS-imides for the CH_4_/CO_2_ and N_2_/CO_2_ gas mixtures at 60 bar. The maximum standard error is 0.4. The yellow lines indicate a α^*^_A/B_ of 1 and the pink lines show the ideal permselectivity for each specific network.

Using the mixed-gas data at 60 bar and at 300 °C and 400 °C, the real solubility *α*^S*^_A/B_ and diffusion *α*^D*^_A/B_ selectivities upon sorption of the 90%/10% mixtures are provided in Table 8 for the four selected networks. The corresponding real separation factors α^*^_A/B_ are displayed in Figure 13. As for Figure 8, the yellow line corresponds to a separation of 1 and for convenience, the ideal permeselectivities *α*_A/B_ have been indicated with pink lines.

Compared to the single-gas feeds (Table 5), the real solubility selectivities *α*^S*^_A/B_ at 300 °C and 400 °C increase in favor of CO_2_ in both mixtures, with the effect being stronger when mixed with N_2_. On the other hand, the real diffusion selectivities *α*^D*^_A/B_ tend to either remain similar (OAPS-based networks) or slightly diminish (POSS-based networks) for the CH_4_/CO_2_ mixture. For the N_2_/CO_2_ mixture, the diffusion selectivity completely disappears. These mixed-gas selectivities under harsh conditions, thus, seem to be mostly governed by the solubility. At low temperatures, it has been shown that the CO_2_/CH_4_ solubility selectivity outweighs the diffusion selectivity in glassy polymers when mixed-gas permeation experiments are carried out, whereas it is the contrary under single-gas conditions [13,119]. In the present case, it follows the same order as for the pure gases, but the ratio between *α*^S*^_A/B_ and *α*^D*^_A/B_ does increase under mixed-gas conditions. The real separation factors are similar to the ideal permselectivities for the dense polyPOSS-imides, but they appear to improve in both polyOAPS-imide networks (Figure 13). While this improvement should be treated with caution given the errors at such high temperatures (reported in all the Tables and Figures), the important point is that both polyOAPS-imide networks seem to be able to maintain their sieving capabilities above 2 at 400 °C, when contacted with 10%CO_2_-containing binary mixtures. Within this context, the poly*ortho*OAPS-PMDA matrix performs slightly better than the poly*meta*OAPS-6FDA, but these remain in both cases outstanding performances for polymer-based membranes. Following this molecular-level screening, it could, thus, be worth further investigating the silane-precursor route for the synthesis of OAPS [49] in order to produce isomer-specific polyOAPS-imides.

## 6. Conclusions

This work highlights the interest of using GCMC-MD and MD atomistic simulations prior to experiments in order to pre-screen candidate membrane structures for gas separation under harsh conditions of temperature and pressure. The sieving properties of a series of networks based on inorganic silsesquioxane POSS or OAPS hyper-cross-linked with small organic PMDA or 6FDA imides have been successfully compared. From an experimental point-of-view, the aliphatic linkers in POSS have been shown to degrade above 300 °C, while the phenyl linkers in OAPS can resist well above 400 °C. The latter is, thus, expected to significantly increase the thermoresistance of the hybrid networks. However, OAPS has three possible *meta*, *para* or *ortho* isomers, which depend on the position of the -NH_2_ group on the phenyl ring and lead to various network connectivities.

Since measurements at high temperatures and pressures are difficult to carry out in a laboratory, eight polyOAPS/POSS-imide model networks have been tested for their N_2_, CH_4_ and CO_2_ permselectivities at 300 °C (for all of them) and at 400 °C (for the polyOAPS-imides only). The feed gas pressure was set to 60 bar in all 54 systems. The network+gas models were first analyzed under single-gas conditions, but to be closer to the real processes, the four networks that maintained CO_2_/CH_4_ and CO_2_/N_2_ ideal permselectivities above 2 at 300 °C and 400 °C were also tested with 90%/10% binary-gas CH_4_/CO_2_ and N_2_/CO_2_ feeds.

In the pure state, the densities and structures of the networks depend on the nature of their precursors. The more open matrices were either based on OAPS or/and PMDA, and the proportions of available free volume covered a large range, i.e., ~2–20%. However, all of them were able to maintain a rather high cohesion at both 300 °C and 400 °C, which suggested that some could preserve their sieving properties. The iterative GCMC-MD procedure was used to load the matrices with gas. Convergence was attained in fewer iterations than at lower temperatures and, in spite of the decrease in solubilities, there were still enough sorbed penetrants for the results to be statistically significant. Similarly, the long-time Fickian diffusive limit could be attained within the MD timescale at such elevated temperatures.

Under single-gas conditions, the *S*_gas_ varied as expected in the same order as the critical temperatures, but the differences between the penetrants were attenuated because of the decrease in the densities. Consequently, the volume swellings were small and the plasticizing effect of CO_2_ was strongly reduced. The *D*_gas_ correlated to the amount of available free volume. In terms of *P*_gas_, the networks could be separated into the following two categories: (i) three OAPS-based networks, which exhibited a nanoporous behavior, and (ii) five networks, including the poly*ortho*OAPS-PMDA, poly*meta*OAPS-6FDA and both polyPOSS-imides, which were less permeable. The four last systems showed the highest ideal CO_2_/CH_4_ and CO_2_/N_2_ permselectivities and, as such, they were further tested with mixed-gas feeds.

Under binary-gas 90%/10% CH_4_/CO_2_ and N_2_/CO_2_ conditions, CO_2_ sorbed more than its percentage in the gas phase and its *S*_gas-mix_ slightly improved. However, this effect decreased with increasing temperature and there were no plasticization effects. The small variations in the *D*_gas-mix_ led to similar diffusion selectivities for the CO_2_/CH_4_ pair, but to a loss of diffusion selectivity for the CO_2_/N_2_ pair. The *P*_gas-mix_ confirmed that such gas separations under harsh conditions are mostly governed by the solubility. The real CO_2_/CH_4_ and CO_2_/N_2_ separation factors were either similar or slightly improved with respect to the ideal permselectivities. Both polyOAPS-imide networks under study seemed to be able to maintain their sieving abilities above 2 at 400 °C, with the poly*ortho*OAPS-PMDA matrix performing slightly better than the poly*meta*OAPS-6FDA. Since these are outstanding performances for polymer-based membranes, the isomer-specific synthesis route for OAPS should, thus, be further investigated in order to subsequently be able to produce isomer-specific polyOAPS-imides using the interfacial polymerization technique.

## Figures and Tables

**Table 1:** Connectivities of the polyOAPS/POSS-imide network models. More details are provided in Refs [59,60].

Connectivities	*meta* OAPS + PMDA	*para* OAPS + PMDA	*ortho* OAPS + PMDA	POSS + PMDA	*meta* OAPS + 6FDA	*para* OAPS + 6FDA	*ortho* OAPS + 6FDA	POSS + 6FDA
Total no. of atoms	33,672	33,828	33,492	31,644	43,183	43,245	41,974	41,362
<No. of links *per* cage>	5.3	5.4	5.2	5.1	4.9	4.9	4.5	4.9
<No. of intercage links *per* cage>	4.6	5.1	3.3	4.3	3.7	3.7	3.9	4.1
<No. of intracage links *per* cage>	0.7	0.3	1.9	0.8	1.2	1.2	0.6	0.8
<No. of different cages linked to a cage> (=cross-linking density)	4.2	4.7	3.0	4.0	3.4	3.5	3.6	3.7

**Table 2:** Average percentages of significant volume <*%FSV*> in the pure polyOAPS/POSS-imides matrices available for the insertion of N_2_, CH_4_ and CO_2_.

<%FSV>	meta OAPS + PMDA	para OAPS + PMDA	ortho OAPS + PMDA	POSS + PMDA	meta OAPS + 6FDA	para OAPS + 6FDA	ortho OAPS + 6FDA	POSS + 6FDA
N_2_ at 300 °C	10.5	19.9	5.2	3.2	6.4	8.9	8.9	2.9
N_2_ at 400 °C	11.6	22.0	5.8	-	7.3	10.2	9.6	-
CH_4_ at 300 °C	8.8	17.8	4.2	2.4	5.1	7.4	7.6	2.1
CH_4_ at 400 °C	9.8	19.8	4.7	-	5.9	8.5	8.2	-
CO_2_ at 300 °C	7.8	16.4	3.6	2.0	4.4	6.5	6.9	1.8
CO_2_ at 400 °C	8.8	18.5	4.2	-	5.1	7.6	7.5	-

**Table 3:** Converged numbers of N_2_, CH_4_ and CO_2_ molecules sorbed by the polyOAPS/POSS-imides networks at both 300 °C and 400 °C and at a single-gas feed pressure of 60 bar. The standard errors on the mean number of penetrants predicted are, in all cases, no more than ±1 molecule (Figure S4, Supporting Information).

No. of Molecules	*meta* OAPS + PMDA	*para* OAPS + PMDA	*ortho* OAPS + PMDA	POSS + PMDA	*meta* OAPS + 6FDA	*para* OAPS + 6FDA	*ortho* OAPS + 6FDA	POSS + 6FDA
N_2_ at 300 °C	255	399	106	73	221	264	187	95
N_2_ at 400 °C	182	287	73	-	147	191	131	-
CH_4_ at 300 °C	346	524	140	97	306	370	246	122
CH_4_ at 400 °C	230	355	93	-	186	243	158	-
CO_2_ at 300 °C	704	943	287	234	610	727	442	281
CO_2_ at 400 °C	411	562	153	-	356	433	259	-

**Table 4:** Average percent of volume swelling upon sorption of N_2_, CH_4_ and CO_2_ penetrants in the polyOAPS/POSS-imides networks at both 300 °C and 400 °C and at 60 bar. The maximum standard error is 0.3%.

% Volume Swelling	*meta* OAPS + PMDA	*para* OAPS + PMDA	*ortho* OAPS + PMDA	POSS + PMDA	*meta* OAPS + 6FDA	*para* OAPS + 6FDA	*ortho* OAPS + 6FDA	POSS + 6FDA
N_2_ at 300 °C	0	1.4	0.4	0.1	0.3	0.2	0.7	−0.2
N_2_ at 400 °C	1.3	1.2	0.1	-	−0.1	1.0	0.2	-
CH_4_ at 300 °C	0.7	2.6	0.5	0.4	1.1	1.4	0.6	0.3
CH_4_ at 400 °C	2.2	1.6	0.5	-	0	1.4	0.2	-
CO_2_ at 300 °C	2.3	3.8	0.9	1.5	1.7	2.6	1.2	1.7
CO_2_ at 400 °C	2.5	2.3	0.3	-	1.3	3.0	0.8	-

**Table 5:** Ideal solubility and diffusion selectivities for the CO_2_/CH_4_ and CO_2_/N_2_ gas pairs in the polyOAPS/POSS-imides at both 300 °C and 400 °C and at 60 bar. The maximum standard error is less than 0.1 for all the selectivities.

*α*^S^_A/B_ and *α*^D^_A/B_	*meta* OAPS + PMDA	*para* OAPS + PMDA	*ortho* OAPS + PMDA	POSS + PMDA	*meta* OAPS + 6FDA	*para* OAPS + 6FDA	*ortho* OAPS + 6FDA	POSS + 6FDA
*α*^S^_CO_2_/CH_4__ at 300 °C	2	1.8	2	2.4	2	1.9	1.8	2.3
*α*^S^_CO_2_/CH_4__ at 400 °C	1.8	1.6	1.6	-	1.9	1.8	1.6	-
*α*^D^_CO_2_/CH_4__ at 300 °C	0.8	0.6	1.5	2	1.2	1	0.9	2.2
*α*^D^_CO_2_/CH_4__ at 400 °C	0.9	0.7	1.6	-	1.1	0.9	0.9	-
*α*^S^_CO_2_/N_2__ at 300 °C	2.7	2.3	2.7	3.2	2.7	2.7	2.4	2.9
*α*^S^_CO_2_/N_2__ at 400 °C	2.2	1.9	2.1	-	2.4	2.2	2	-
*α*^D^_CO_2_/N_2__ at 300 °C	0.8	0.6	1	1.2	0.9	0.9	0.8	1.5
*α*^D^_CO_2_/N_2__ at 400 °C	0.7	0.7	1.1	-	1	0.8	0.9	-

**Table 6:** Converged numbers of N_2_, CH_4_ and CO_2_ molecules sorbed by the polyOAPS/POSS-imides networks at 300°C and 400°C upon contact with binary 90%/10% mixtures of either CH_4_/CO_2_ or N_2_/CO_2_ at 60 bar.

No. of Molecules	*ortho* OAPS + PMDA	POSS + PMDA	*meta* OAPS + 6FDA	POSS + 6FDA
CH_4_/CO_2_ at 300 °C	125/35	91/27	259/78	105/32
CH_4_/CO_2_ at 400 °C	77/16	-	166/38	-
N_2_/CO_2_ at 300 °C	93/36	68/30	193/85	82/34
N_2_/CO_2_ at 400 °C	63/17	-	128/40	-

**Table 7:** Average percent of volume swelling upon sorption of 90%/10% CH_4_/CO_2_ and N_2_/CO_2_ mixtures at 60 bar in the four selected polyOAPS/POSS-imides at both 300 °C and 400 °C. The maximum standard error is 0.2%.

% Volume Swelling	*ortho* OAPS + PMDA	POSS + PMDA	*meta* OAPS + 6FDA	POSS + 6FDA
CH_4_/CO_2_ at 300 °C	0.6	0.2	0.6	0.1
CH_4_/CO_2_ at 400 °C	0.2	-	0.5	-
N_2_/CO_2_ at 300 °C	0.3	0.2	1.2	−0.2
N_2_/CO_2_ at 400 °C	0	-	−0.2	-

**Table 8:** Real solubility and diffusion selectivities for the CO_2_/CH_4_ and CO_2_/N_2_ gas pairs in the selected polyOAPS/POSS-imides, at both 300 °C and 400 °C and at 90%/10% CH_4_/CO_2_ and N_2_/CO_2_ feed pressures of 60 bar. The maximum standard error is less than 0.2 for all the selectivities.

*α*^S*^_A/B_ and *α*^D*^_A/B_	*ortho* OAPS + PMDA	POSS + PMDA	*meta* OAPS + 6FDA	POSS + 6FDA
*α*^S*^_CO_2_/CH_4__ at 300 °C	2.5	2.7	2.7	2.7
*α*^S*^_CO_2_/CH_4__ at 400 °C	1.9	-	2.1	-
*α*^D*^_CO_2_/CH_4__ at 300 °C	1.4	1.7	1.3	1.7
*α*^D*^_CO_2_/CH_4__ at 400 °C	1.7	-	1.2	-
*α*^S*^_CO_2_/N_2__ at 300 °C	3.5	4	4	3.7
*α*^S*^_CO_2_/N_2__ at 400 °C	2.4	-	2.8	-
*α*^D*^_CO_2_/N_2__ at 300 °C	0.9	1	1	1.1
*α*^D*^_CO_2_/N_2__ at 400 °C	1	-	0.9	-

## Data Availability

Data are available on demand to the authors.

## Supplementary Materials

The following supporting information can be downloaded at: https://www.mdpi.com/article/10.3390/membranes12050526/s1, Figure S1: synthesis of the polyOAPS-imides; Figure S2: partial charges for all OAPS-based networks; Figure S3: radial distribution functions in the mixtures; Figure S4: variations in the number of sorbed CO_2_ at 60 bar predicted by GCMC for successive configurations at 300 °C; Figure S5: typical interOAPS-imide and intraOAPS-imide links. Figure S6: probability density distributions for the number of arms linked per cage in the networks. Table S1: atom-types for all OAPS-based molecules; Table S2: force-field parameters for the bonds, bending and out-of-plane potentials; Table S3: force-field parameters for the torsional and van der Waals potentials; Table S4: concentrations and solubilities of the gases in the pure gas phase at 300 °C and 400 °C and at a pressure of 60 bar; Table S5: concentrations and solubilities of the binary mixtures in the mixed-gas phase at 300 °C and 400 °C and at a pressure of 60 bar.
