# Supplementary material for: Molecular Characterization of Membrane Gas Separation under Very High Temperatures and Pressure: Single- and Mixed-Gas CO2/CH4 and CO2/N2 Permselectivities in Hybrid Networks
Source: Membranes (Basel). 2022 May 17;12(5):526. doi: 10.3390/membranes12050526 (PMC9143592; doi:10.3390/membranes12050526)
Supplement: Supplementary file 1 [file membranes-12-00526-s001.zip › membranes-1706960-supplementary.pdf]

SUPPLEMENTARY INFORMATION

Molecular characterization of membrane gas separation under very high temperatures and pressure: single- and mixed-gas CO_2/CH_4 and CO_2/N_2 permselectivities in hybrid networks

Sylvie Neyertz, David Brown, Saman Salimi, Farzaneh Radmanesh and Nieck E. Benes

1) THE EXPERIMENTAL PROCEDURE

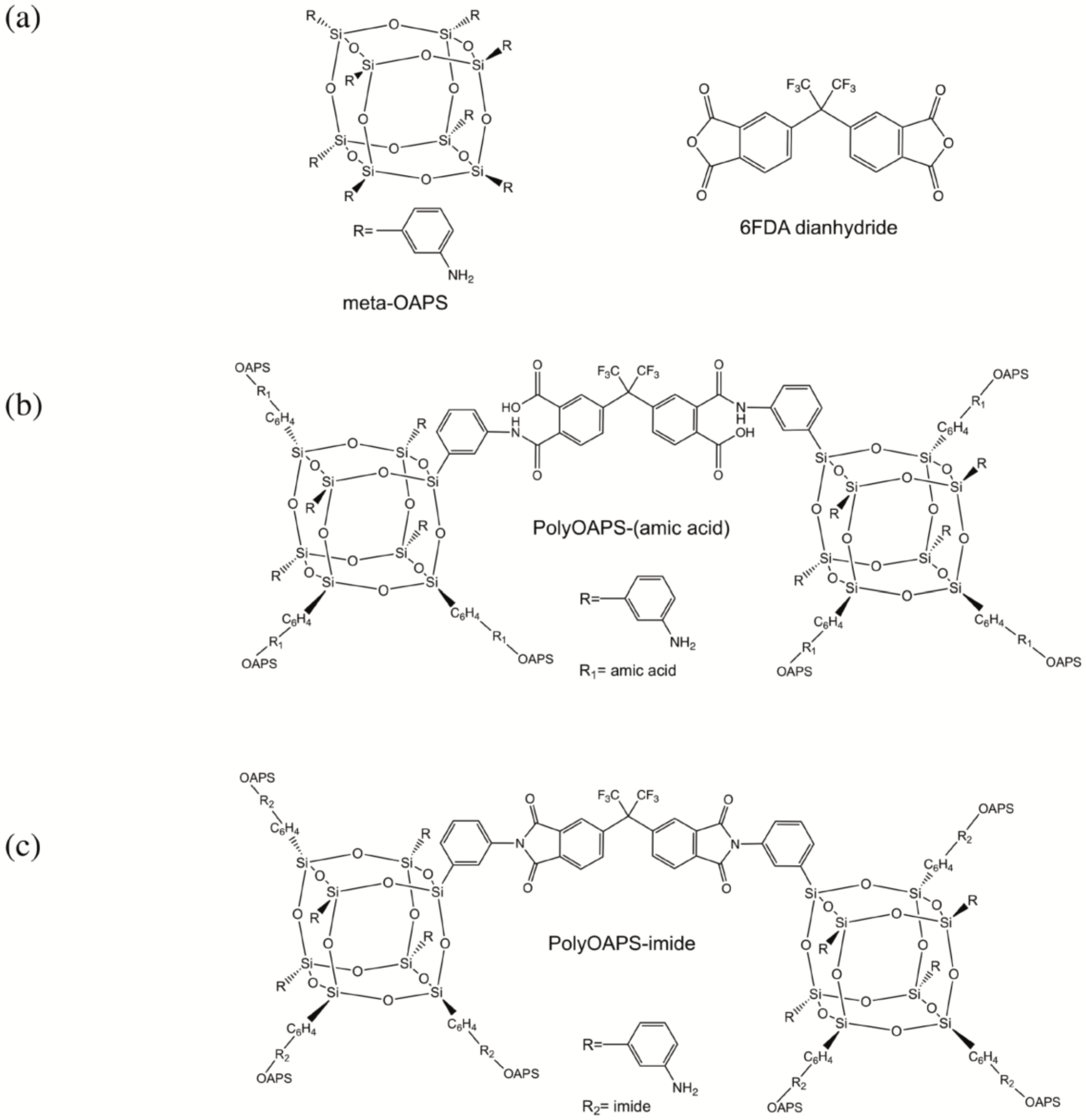

Figure S1 Schematic representation of the synthesis of the hyper-cross-linked polyOAPS-imide networks based on the *meta*OAPS and the 6FDA precursors. **(a)** the reactants, **(b)** the interfacial polycondensation and **(c)** the thermal imidization carried out at 300°C.

2) ATOM-TYPES AND FORCE-FIELD PARAMETERS

The atom-types used for OAPS and the polyOAPS-imides are defined in Table S1. The atom-types and the parameters for the aliphatic POSS and polyPOSS-imides have been given previously.

Table S1 Atom-types for all OAPS-based molecules

Atom-type	Description
Si	Silicon on siloxane cage
O	Oxygen on siloxane cage
Car	Aromatic carbon
Hcar	Hydrogen on aromatic carbon
Npri	Nitrogen on primary amine
Hnpri	Hydrogen on primary amine
Ckanhy	Ketone carbon on dianhydride
Okanhy	Ketone oxygen on dianhydride
Oanhy	Anhydride oxygen on dianhydride
Ckimide	Ketone carbon on polyOAPS-imide
Okimide	Ketone oxygen on polyOAPS-imide
Nimide	Imide nitrogen on polyOAPS-imide
C_1	$(CF_3)_2$-bearing carbon on 6FDA dianhydride and polyOAPS-imide
C_2	F_3-bearing carbon on 6FDA dianhydride and polyOAPS-imide
F	Fluorine on 6FDA dianhydride and polyOAPS-imide
Oether	Ether oxygen on ODPA dianhydride and polyOAPS-imide

The classical force-field used for the MD simulations in the *gmq* code is given by Equation A:

$$U_{pot} = \sum_{\theta} U_{bend}(\theta) + \sum_{\tau} U_{tors}(\tau) + \sum_{i-planar} U_{oop}(i) + \sum_{(i,j)nb} U_{vdw}(r) + \sum_{(i,j)nb} U_{coul}(r) \qquad \text{(A)}$$

where the first three terms are referred to as the "bonded" potentials and the last two terms as the "nonbonded" potentiels. All bonds were kept rigid using the SHAKE algorithm.The five terms in Equation A are detailed hereafter.

The angle-bending deformations are described by a harmonic function in the cosine of the bond angles θ:

$$U_{bend}(\theta) = \frac{k_\theta}{2}\left(\cos\theta - \cos\theta_0\right)^2 \tag{B}$$

where k_θ is a constant determining the flexibility of the angle and θ_0 is the equilibrium bond angle. No specific angle-bending potentials were used to restrict the Si-O-Si and O-Si-O angles, since the combination of the rigid Si-O bonds and the non-bonded U_{vdw} and U_{coul} terms were sufficient to maintain the geometry of the cage. To ensure the equipartition of kinetic energy, the high-frequency motions of hydrogens in CH_2, CH and NH_2 groups were removed using special constraints. The equilibrium Hnpri-Npri-Hnpri and Car-Npri-Hnpri angles were set to 112.2 and 115.4 degrees, respectively.

The torsional motions around the dihedral angles τ are represented by a third-order polynomial in $\cos\tau$:

$$U_{tors}(\tau) = \sum_{n=0}^{3} a_n \cos^n \tau \tag{C}$$

with the dihedral angle τ varying from -180° to +180°, $\tau = 0°$ being the *trans* conformation and a_n being the torsional coefficients. The wild card denotes any atom-type, except when the coefficients for a specific dihedral are defined elsewhere. It should be noted that the *-Si-Car-* dihedral potential had to be represented by a sixth-order polynomial, so the sum in Eq. C runs from $n = 0$ to 6 for that specific angle.

The out-of-plane term, keeps sp^2 structures planar by using a harmonic function in the perpendicular distance d from the central atom i to the plane defined by its three attached atoms with k_{oop} being the force constant:

$$U_{oop}(i) = \frac{k_{oop}}{2} d^2 \tag{D}$$

A coplanar vector bisector constraint designed to remove the degrees of freedom of the Hcar atoms in the aromatic Car-Hcar groups is applied as a special form of the out-of-plane terms.

The "nonbonded" excluded-volume van der Waals and electrostatic potentials, which depend on the distance r between two interacting sites, were applied to all atom pairs situated either on the same molecule (but separated by more than two bonds if the intervening angles are subject to the bending potential or by more than one bond otherwise) or on two different molecules. The van der Waals interactions are described in Equation A by the 12-6 Lennard-Jones form:

$$U_{vdw}(r) = U_{LJ}(r) = 4\varepsilon\left(\left(\frac{\sigma}{r}\right)^{12} - \left(\frac{\sigma}{r}\right)^{6}\right) \tag{E}$$

where ε is the well-depth of the potential and σ is the distance at which the potential is zero. The cross-terms for unlike-atom pairs are obtained from the standard combination rules given by:

$$\sigma_{ij} = \frac{\sigma_{ii} + \sigma_{jj}}{2} \qquad \varepsilon_{ij} = \sqrt{\left(\varepsilon_{ii} \times \varepsilon_{jj}\right)} \tag{F}$$

The van der Waals cutoff was set to 12 Å and long-range corrections to the energy and the pressure were implemented.

The last term of Equation A accounts for the Coulombic interactions with q_i and q_j being the partial charges on atoms *i* and *j* respectively, and ε_0 being the vacuum permittivity:

$$U_{coul}\left(r\right) = \frac{q_i q_j}{4\pi\varepsilon_0 r} \tag{G}$$

This long-range electrostatic potential was calculated using the Ewald summation method, with a real space cutoff R_c of 12 Å, the reciprocal space sum K_{max} being typically equal to 12-14 and the separation parameter α being typically equal to 0.19-0.23 depending on the systems under study.

The average bond-lengths b_0, bond-angles θ_0 and partial charges q_i/e were obtained by performing Density Functional Theory (DFT) optimizations on representative fragments of the molecules under study in vacuum with the Gaussian09 code at the B3LYP/6-31G** level. The partial charges were then extracted by an ESP-fitting procedure and symmetrized with respect to the nature and the position of the atoms. The partial charges on the unreacted $-NH_2$ arms of the cross-linked OAPS were kept identical to those in the pure OAPS, since they were very close in the DFT-optimised systems.

Similarly, the *-Si-Car-* and the *-Npri-Car-* torsional potentials were obtained from the DFT optimizations by calculating the total energy of the *para*OAPS molecule as a function of the rotation around these specific bonds, and adapting it to the form of the present classical force-field (Eq. A). Because of the symmetry of the tetrahedral Si and the planar Car, a sixth-order polynomial in the cosine of the torsional angle had to be used in place of the third-order polynomial of Eq. C for the *-Si-Car* potential. Only its a_0 and a_6 (indicated by a hash sign # in Table S3) coefficients were non-zero. On the other hand, the *-Npri-Car-* torsional potential could be described by Eq. C.

The other force-field parameters were adapted from the literature.[13-15] As used elsewhere for aromatic siloxanes, the O-Si-Car bending constant was chosen equal to the corresponding one for Si and aliphatic C. All parameters used in Equation A are given in Tables S2-S3 and in Figure S2.

Table S2 Force-field parameters for the bonds, bending (Eq. B) and out-of-plane (Eq. D) potentials. The symbols - show that no parameters are defined for these interactions.

**Bond types**	b_0 / Å	**Bond-angle type**	θ_0 / deg	k_θ / kJ.mol^{-1}	**Out-of-plane atom**	k_{oop} / kg s^{-2}
Si-O	1.65	O-Si-O	109.0	-	Car	667
Si-Car	1.85	Si-O-Si	148.8	-	Ckanhy	667
Car-Car	1.40	O-Si-Car	109.9	477.8	Ckimide	667
Car-Npri	1.39	Si-Car-Car	120.9	879.1	Nimide	167
Car-Nimide	1.43	Car-Car-Car	120.0	879.1		
Car-Ckanhy	1.49	Car-Car-Npri	120.6	1465.1		
Car-Ckimide	1.49	Car-Car-Nimide	119.6	2270.9		
Car-Oether	1.38	Car-Car-Ckanhy	119.0	879.1		
Car-C1	1.55	Car-Car-Ckimide	119.1	879.1		
Car-Hcar	1.08	Car-Car-Oether	119.1	2270.9		
Ckanhy-Okanhy	1.20	Car-Car-C_1	120.2	879.1		
Ckanhy-Oanhy	1.40	Car-Car-Hcar	120.1	879.1		
Ckimide-Okimide	1.21	Car-Nimide-Ckimide	124.2	1904.6		
Ckimide-Nimide	1.42	Car-Ckanhy-Okanhy	130.3	952.3		
Npri-Hnpri	1.01	Car-Ckanhy-Oanhy	107.1	1098.8		
C_1-C_2	1.56	Car-Ckimide-Okimide	128.2	952.3		
C_2-F	1.35	Car-Ckimide-Nimide	105.7	1465.1		
		Car-Oether-Car	121.5	622.2		
		Car-C_1-Car	111.4	556.5		
		Car-C_1-C_2	109.4	742.0		
		Okanhy-Ckanhy-Oanhy	122.6	1098.8		
		Ckanhy-Oanhy-Ckanhy	110.4	622.2		
		Okimide-Ckimide-Nimide	126.1	1171.7		
		Ckimide-Nimide-Ckimide	111.5	659.3		
		C_2-C_1-C_2	108.0	742.0		
		C_1-C_2-F	111.5	618.3		
		F-C_2-F	107.4	1236.6		

Table S3 Force-field parameters for the torsional (Eq. C) and van der Waals (Eq. E) potentials. The symbols * denote a wild card. See text for detail for the hash sign #

**Dihedral type**	a_0 / kJ.mol^{-1}	a_1 / kJ.mol^{-1}	a_2 / kJ.mol^{-1}	a_3 or $a_6^{\#}$/ kJ.mol^{-1}	**Like-atom pair**	σ / Å	ε_{ii}/k_B / K
-Si-Car-	-0.135	0	0	$0.480^{\#}$	Si...Si	3.385	294.38
-Car-Car -	16.736	0	-16.736	0	O...O	2.955	102.15

-Car-Npri-	11.380	0	-11.380	0
-Car-Nimide-	13.389	0	-13.389	0
-Car-Ckanhy-	13.389	0	-13.389	0
-Car-Ckimide-	13.389	0	-13.389	0
-Car-Oether-	10.041	0	-10.041	0
-Car-C_1-	0.502	-1.506	0	2.008
-Ckanhy-Oanhy-	48.534	0	-48.534	0
-Ckimide-Nimide-	54.057	0	-54.057	0
*-C_1-C_2-F	0.837	2.510	0	-3.347

Car...Car	3.029	53.84
Ckanhy...Ckanhy	3.029	53.84
Ckimide...Ckimide	3.029	53.84
C_1...C_1	3.029	53.84
C_2...C_2	3.029	53.84
Oanhy...Oanhy	2.708	58.37
Okanhy...Okanhy	2.708	58.37
Okimide...Okimide	2.708	58.37
Oether...Oether	2.708	58.37
Npri...Npri	2.762	47.81
Nimide...Nimide	2.762	47.81
Hcar...Hcar	2.673	21.14
Hnpri...Hnpri	2.673	21.14
F...F	2.619	54.85

poly*meta*OAPS-6FDA

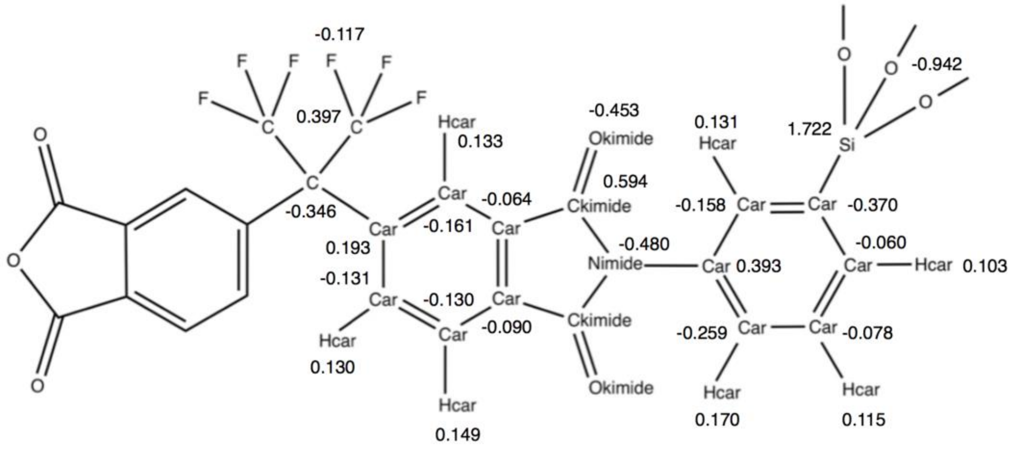

poly*para*OAPS-6FDA

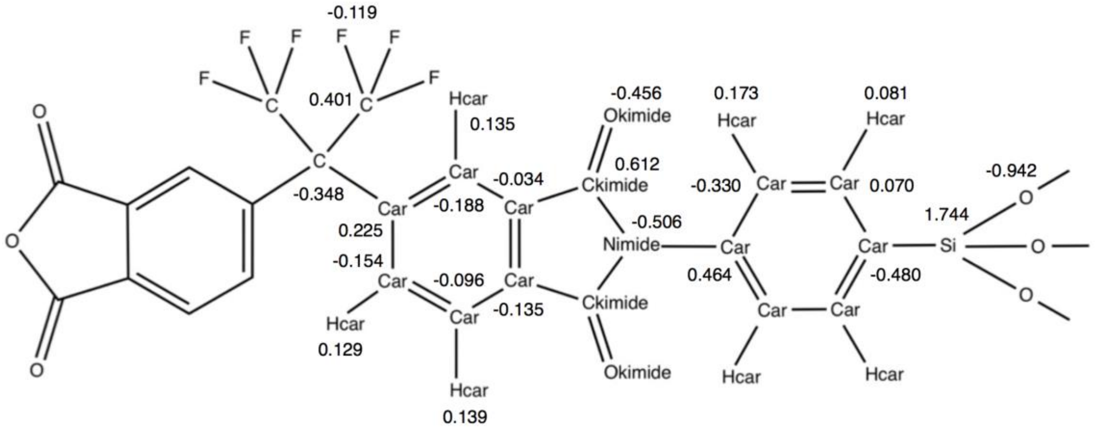

poly*ortho*OAPS-6FDA

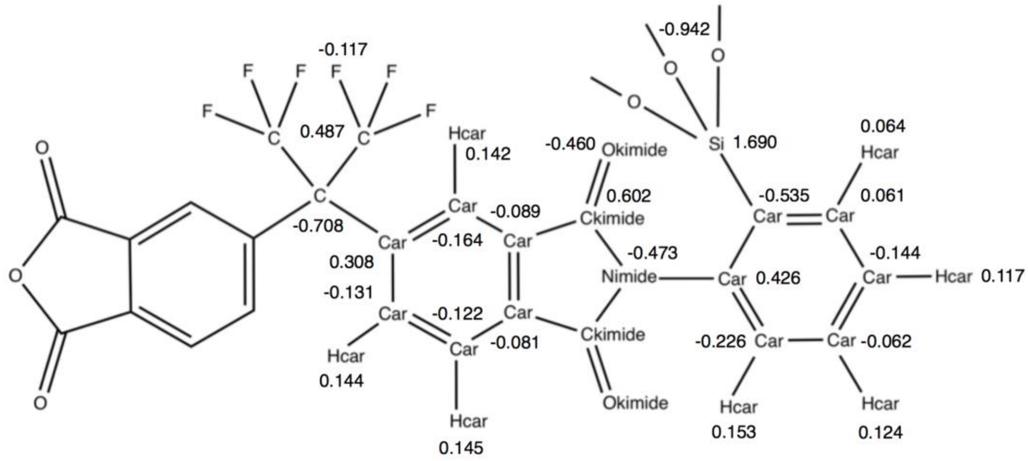

poly*meta*OAPS-PMDA

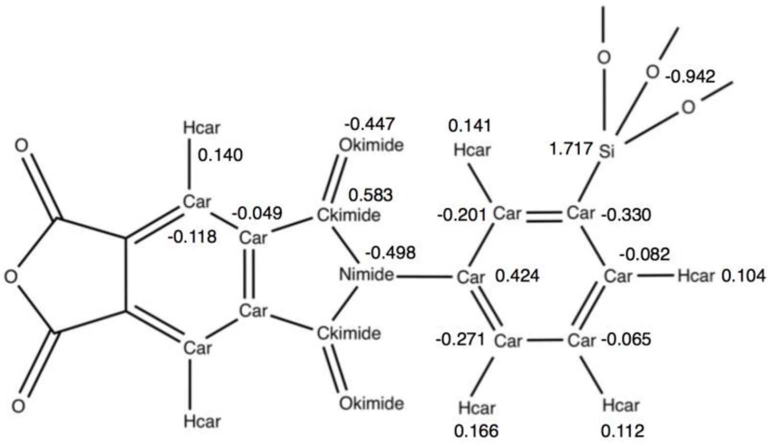

poly*para*OAPS-PMDA

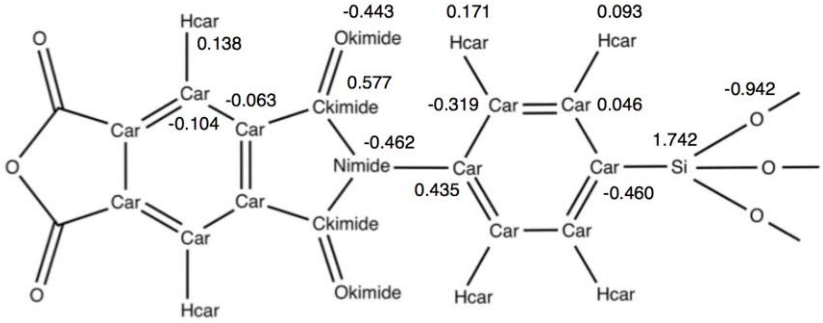

poly*ortho*OAPS-PMDA

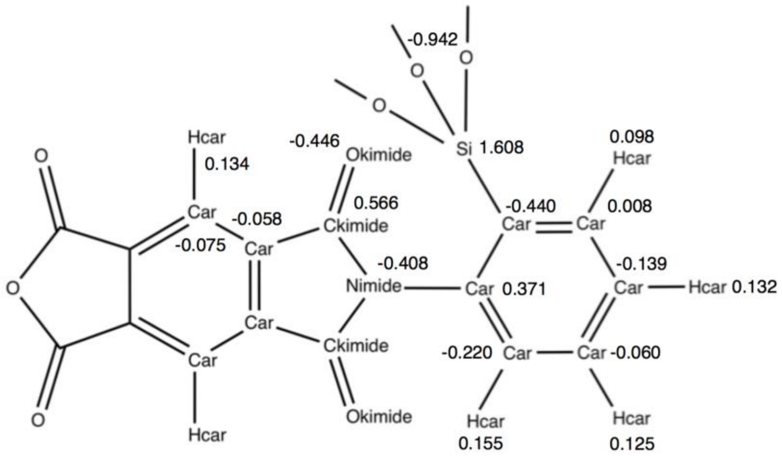

Figure S2 The partial charges q_i/e for all OAPS-based networks.

3) CHOICE OF THE LIMIT CRITERION FOR CROSS-LINKING

The limit criterion, which controls which dianhydrides are selected for cross-linking, is based on the mixtures radial distribution functions g(*r*) between the OAPS amine nitrogen N and the dianhydride ketone carbons C_{ket}. Only those dianhydrides having the sum, R_{min}, of their shortest C_{ket}···N distances at either end being less than the limit criterion are reacted. The C_{ket}···N g(*r*) are displayed in Figure S3. Unlike the *para*OAPS and *meta*OAPS isomers, there are relatively few cases in the dianhydride:*ortho*OAPS mixture, where the C_{ket}···N distances are less than 3 Å. Using a limit criterion of 6 Å (as was done before for polyPOSS-PMDA) would thus exclude all connections via the *ortho*-substituted OAPS. Without any experimental information suggesting that imide bridges between *ortho*-substituted arms are unlikely, the criterion for reactions was set to $R_{min} \leq 7$ Å for both polyOAPS-imide and polyPOSS-imide networks. This was shown to give identical results to the initial R_{min} of 6 Å.

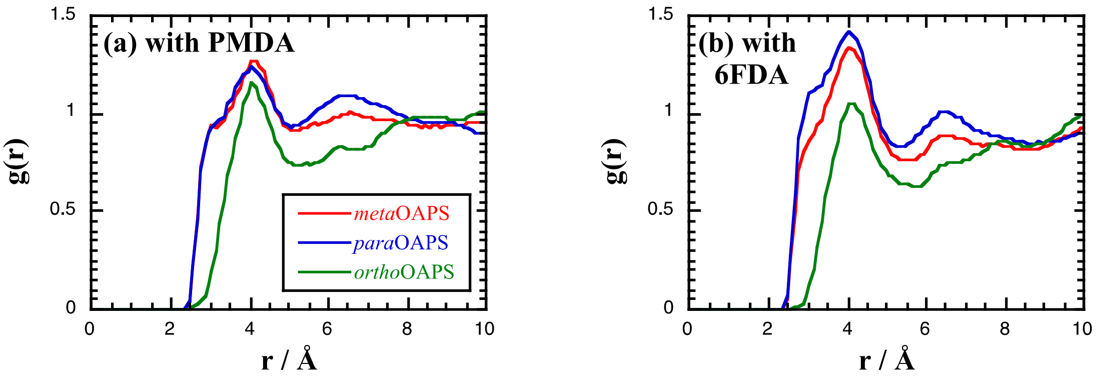

Figure S3 Radial distribution functions g(*r*) in the mixtures between the dianhydride C_{ket} and the OAPS N as a function of the nature of the dianhydride and the OAPS isomer.

4) PURE GAS SIMULATIONS

For the gases in the pure gas phase, MD simulations were carried out under the same conditions than the network+gas models on single-gas systems containing either 1000 molecules of N_2, 512 molecules of CH_4 or 512 molecules of CO_2. For the 90%/10% CH_4+CO_2 and N_2+CO_2 binary-gas mixtures, the systems also contained 1000 molecules in total, but with 900 molecules of either CH_4 or N_2 and 100 molecules of CO_2. The systems were relaxed at 60 bar up to 10000 ps under constant isotropic-pressure *NpT* conditions at both 300°C and 400°C. Averages of the gas solubilities S_{feed} in the pure gas phase were obtained using test particle insertion (TPI) methods over the last 5000 ps of the simulations. The average gas phase concentrations C_{feed} at p = 60 bar were determined directly from the relaxed MD simulations. The C_{feed} and S_{feed} are provided in Table S4 for the single-gas systems and in Table S5 for the binary-gas systems.

Table S4 Concentrations and solubilities of methane, carbon dioxide and nitrogen in the pure gas phase at 300°C and 400°C and at a pressure of 60 bar as used in the GCMC calculations to define the single-gas feed characteristics.

T / °C	Gas	C_{feed} / Molecules/nm^3	S_{feed}
**300**	**CH_4**	0.74453	0.96227
	**CO_2**	0.76876	1.02802
	**N_2**	0.73760	0.94649
**400**	**CH_4**	0.63116	0.95612
	**CO_2**	0.64480	0.99848
	**N_2**	0.62967	0.94771

Table S5 Concentrations and solubilities of binary 90%/10% mixtures of methane+carbon dioxide and nitrogen+carbon dioxide in the mixed-gas phase at 300°C and 400°C and at a pressure of 60 bar as used in the GCMC calculations to define the mixed-gas feed characteristics.

**Binary Mixture**	**90%/10% CH_4+CO_2**				**90%/10% N_2+CO_2**			
**T / °C**	C_{CH4} / Molec./nm^3	C_{CO2} / Molec./nm^3	S_{CH4}	S_{CO2}	C_{N2} / Molec./nm^3	C_{CO2} / Molec./nm^3	S_{N2}	S_{CO2}
**300**	0.67114	0.07457	0.96445	0.98922	0.66653	0.07406	0.94873	0.97673
**400**	0.56993	0.06333	0.95750	0.97383	0.56710	0.06301	0.94919	0.96660

5) CONVERGENCE OF GCMC SIMULATIONS

Figure S4 shows the initial GCMC step for CO_2 uptake in the pure poly*para*OAPS-PMDA network at 300°C. The numbers of carbon dioxide molecules absorbed into the systems are plotted (thin coloured lines) as a function of the number of MC moves for all 20 different configurations used. The thick black line gives the average result. The initial rise towards a plateau value is relatively rapid at these elevated temperatures. It can also be seen that the numbers of molecules absorbed in each system fluctuate around the average value with relatively modest deviations (maximum of about ±10% of the mean value). There are no obvious systematic differences from configuration to configuration and the fluctuations in the mean value are sufficiently limited to obtain a reliable estimate of the number of absorbed penetrant molecules to include for the subsequent MD step. Indeed, the standard errors on the mean number of penetrants predicted in the GCMC phase are, in all cases, no more than ±1 molecule. Standard deviations in the mean are all less than 6 molecules.

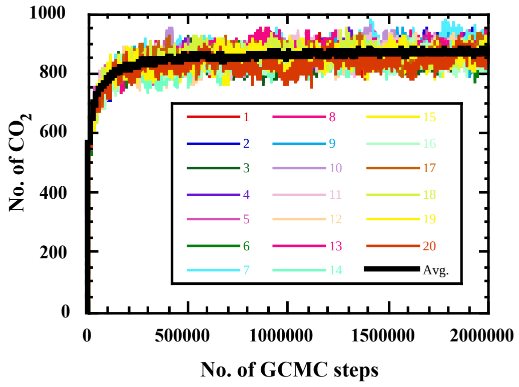

Figure S4 Variations in the number of sorbed CO_2 at 60 bar predicted by GCMC for 20 successive configurations of the pure poly*para*OAPS-PMDA network at 300°C (thin lines with different colours). The average number of sorbed CO_2 is indicated by the thick black line.

6) NETWORK CONNECTIVITIES

Hyper-cross-linked networks based on either OAPS or POSS cages are very complex. Their organic-inorganic links include dianhydrides being linked to two different cages, which are referred to as "intercage-imide" links. However, imides can also be attached to two arms of the same cage, which are referred to as "intracage-imide" links. A representative subset of interOAPS-imide and intraOAPS-imide links is provided in Figure S5.

	**interOAPS-imide links**	**intraOAPS-imide links**
**poly*meta*OAPS-6FDA**	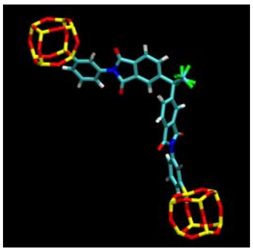	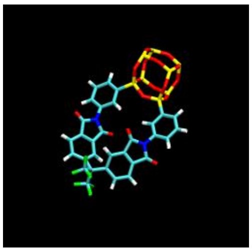
**poly*para*OAPS-PMDA**	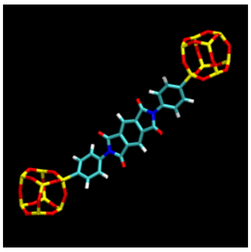	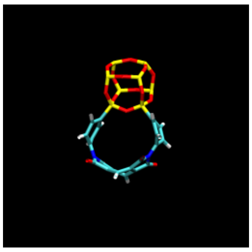

Figure S5 Typical interOAPS-imide and intraOAPS-imide links [19]. Colour code: yellow = Si, red = O, cyan = C, blue = N, green = F, white = H.

The probability distributions of the number of arms linked *per* cage are Gaussian and some of them are presented in Figure S6.

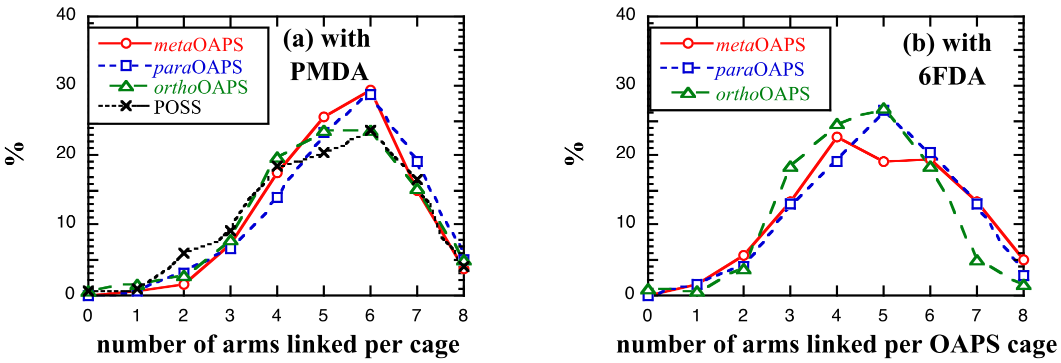

Figure S6 Probability density distributions for the number of arms linked *per* cage in **(a)** the polyOAPS/POSS-PMDA and **(b)** the polyOAPS-6FDA networks.
